# Farnesylated heat shock protein 40 is a component of membrane-bound RISC in *Arabidopsis*

**DOI:** 10.1074/jbc.RA118.003887

**Published:** 2018-09-07

**Authors:** Lars Sjögren, Maïna Floris, Andrea Barghetti, Franziska Völlmy, Rune Linding, Peter Brodersen

**Affiliations:** From the ‡ Department of Biology, University of Copenhagen, Ole Maaløes Vej 5, DK-2200 Copenhagen N and; the § Biotech Research and Innovation Centre, Ole Maaløes Vej 5, DK-2200 Copenhagen N, Denmark

**Keywords:** Arabidopsis thaliana, Argonaute, chaperone DnaJ (DnaJ), microRNA mechanism, membrane, ARGONAUTE, Chaperones, Protein farnesylation, Rough Endoplasmic Reticulum, Small RNA

## Abstract

ARGONAUTE1 (AGO1) binds directly to small regulatory RNA and is a key effector protein of post-transcriptional gene silencing mediated by microRNA (miRNA) and small interfering RNA (siRNA) in *Arabidopsis*. The formation of an RNA-induced silencing complex (RISC) of AGO1 and small RNA requires the function of the heat shock protein 70/90 chaperone system. Some functions of AGO1 occur in association with endomembranes, in particular the rough endoplasmic reticulum (RER), but proteins interacting with AGO1 in membrane fractions remain unidentified. In this study, we show that the farnesylated heat shock protein 40 homologs, J2 and J3, associate with AGO1 in membrane fractions in a manner that involves protein farnesylation. We also show that three changes in AGO1 function are detectable in mutants in protein farnesylation and J2/J3. First, perturbations of the HSP40/70/90 pathway by mutation of *J3*, *HSP90*, and farnesyl transferase affect the amounts of AGO1 associated with membranes. Second, miRNA association with membrane-bound polysomes is increased in farnesyl transferase and farnesylation-deficient J2/J3 mutants. Third, silencing by noncell autonomously acting short interfering RNAs is impaired. These observations highlight the involvement of farnesylated J2/J3 in small RNA-mediated gene regulation, and suggest that the importance of chaperone-AGO1 interaction is not limited to the RISC assembly process.

## Introduction

microRNAs (miRNAs)^4^ and short interfering RNAs (siRNAs)are 20–24-nucleotide small RNAs that exert gene regulation in plants and animals (1). They bind directly to proteins of the ARGONAUTE (AGO) family to form RNA-induced silencing complexes (RISCs) (2, 3), and use base pairing to select specific, complementary mRNA for repression (4). miRNA/siRNA-AGO complexes are assembled in an incompletely understood process termed RISC loading. RISC loading in plants and animals requires the molecular chaperones HSP70 and HSP90 (5–7) and in addition, HSP90 co-chaperones are needed for full miRNA function *in vivo* (8–10). Many native proteins require chaperone-catalyzed conformational changes for biological activity (11), and evidence for the existence of a conserved chaperone assembly line is now emerging based in particular on biochemical and structural studies of mammalian and avian steroid hormone receptors (11, 12). This assembly line is initiated by interaction of the client protein with an HSP40 dimer that transfers the client to HSP70-ATP and catalyzes HSP70 ATPase activity leading to formation of a high-affinity, ternary HSP70-ADP-client complex (13, 14). The co-chaperone Hop then mediates client transfer to HSP90 for conformational maturation together with HSP90 co-chaperones whose identities remain incompletely described (11, 15). Single-molecule observations of encounters between siRNA duplexes and *Drosophila* Ago2 indicates that conformational changes required for RISC loading involve a similar chaperone assembly line, such that chaperone activity opens the conformation of unloaded Ago2 and extends the dwell time of siRNA duplexes on Ago2 to increase the frequency of Ago2-siRNA duplex encounters that result in RISC formation (16, 17). It is unclear whether other chaperone-assisted conformational changes are involved in functions of the mature loaded RISC, but the presence of HSP70 in *Drosophila* Ago1-containing RISC purified using capture oligonucleotides complementary to a specific miRNA suggests that Ago-chaperone interactions may not be confined to the loading process (18).

Early studies of human Ago2 showed that it is a peripheral membrane protein that associates with rough endoplasmic reticulum (RER) and Golgi membranes in a manner dependent on HSP90 activity (19, 20). More recent studies have confirmed AGO association with endomembrane compartments in plants and animals (21–23), and have shown that at least three important AGO functions occur in association with membranes. First, mRNA target repression can occur at the RER. In *Arabidopsis*, the RER is a site of miRNA-guided translational repression and of miRNA-initiated production of phased secondary siRNAs via RNA-dependent RNA polymerase (24, 25), and in human cells, the fraction of RISC active in experimental RNAi was also localized to the RER (26). Second, sorting at multivesicular bodies is important for small RNA activity in both fly and mammalian cells (22, 23), perhaps because of effects on RISC loading and disassembly at these compartments. Third, the membrane-dependent autophagy pathway is employed for regulated proteolysis of AGO proteins in both plants and animals (27, 28). It is unclear which cofactors may be required for these different elements of membrane-associated RISC function, and the mechanism of recruitment of AGO to membrane compartments remains ill-defined in all organisms. Indeed, our knowledge on factors that associate with AGO specifically in membrane compartments is limited in all organisms, although an AGO-interacting nucleoporin localizing to a specific subdomain of the ER was recently shown to be involved in target association of RISC in *Caenorhabditis elegans* (29).

We previously showed that small RNA activity is defective and that membrane association of the main miRNA effector in plants, AGO1, is decreased in *Arabidopsis hmg1* mutants with lesions in the key enzyme in the mevalonate pathway, 3-hydroxy-3-methylglutaryl-CoA reductase (21). 3-Hydroxy-3-methylglutaryl-CoA reductase inhibition or knockdown of other components of the mevalonate pathway in *C. elegans* also led to defective miRNA function (30). The mevalonate pathway produces a cytoplasmic pool of isopentenyldiphosphate that serves as a precursor for several essential lipids (31). These include sterols required for physicochemical properties of biomembranes (32), dolichol required for protein glycosylation (33), and prenyldiphosphate chains required for post-translational modification of proteins (34). Our previous study pointed to the relevance of sterols for miRNA activity in *Arabidopsis* (21), whereas Shi and Ruvkun (30) concluded that dolichol, and hence protein *N*-glycosylation, was particularly important for miRNA activity in *C. elegans*. Nonetheless, both studies indicated that additional groups of isoprenoid metabolites may be relevant for miRNA function (21, 30).

Protein prenylation, either by C_15_H_25_ farnesyl or C_20_H_35_ geranylgeranyl chains, regulates the membrane association and other activities of many proteins (35). Small G-proteins of the Rab family have dedicated prenylation enzymes, whereas prenylation of other proteins require the presence of a Cys-aliphatic-aliphatic-Xaa (C*AAX*) motif at their C terminus (35). C*AAX* farnesyl and geranylgeranyl transferases are heterodimeric enzymes composed of the same α-subunit (PLURIPETALA (PLP) in *Arabidopsis*) and different β-subunits that confer prenyl substrate specificity (36, 37). In *Arabidopsis*, farnesyl transferase contains the β-subunit ENHANCED-RESPONSE-TO-ABSCISIC-ACID1 (ERA1), whereas geranylgeranyl transferase contains the subunit GERANYLGERANYL TRANSFERASE BETA (GGB) (38, 39).

Our recent results have clarified that the requirement for farnesylation of the two closely related HSP40 proteins J2 and J3 explains several phenotypes of farnesyl transferase mutants, and that J2/J3 farnesylation is required for expression of a specific set of abiotic stress-regulated miRNAs (40). In this study, we show that farnesylated J2/J3 associate with AGO1 in membrane compartments and that chaperone function, including J2/J3 farnesylation, influences membrane association of AGO1. The predominant association of AGO1 with ER membranes over other endomembranes is not affected by J2/J3 farnesylation, nor is AGO1 loading with small RNA reduced upon loss of J2/J3 farnesylation. We also find that J2/J3 farnesylation affects the distribution of small RNAs between membrane-bound ribosome-containing heavy fractions and light fractions. These results implicate farnesylated chaperones in functions of AGO1 and small RNAs at membrane compartments.

## Results

### Farnesyl transferase interacts genetically with DICER-LIKE1 (DCL1)

**Figure 1.**
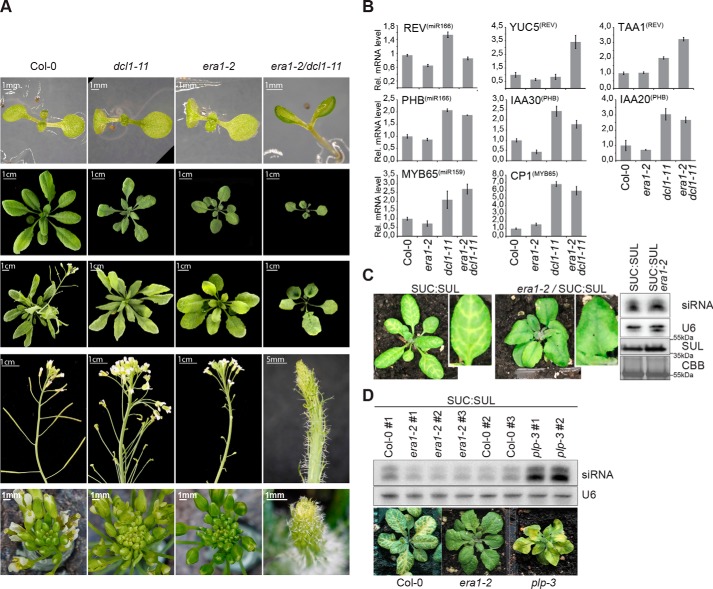
**Farnesyl transferase mutants show defects related to small RNA pathways.** *A,* cotyledon, leaf, and inflorescence phenotypes of Col-0 WT, *era1–2*, *dcl1–11*, and *era1–2/dcl1–11* mutants. *B,* relative mRNA expression levels of three miRNA targets encoding transcription factors (PHB, REV, and MYB65), as well as direct targets of these transcription factors. The figure shows results from a single biological replicate in which RNA from each genotype was prepared from pools of 12 adult leaves. *Error bars* indicate standard error in technical triplicates. Similar results were obtained when the entire experiment was repeated at another point in time. *C, left*, WT and *era1–2* plants expressing the SUC:SUL (SS) hairpin. *Right*, *ChlI* (*SUL*) siRNA and protein levels of the individual plants shown on the *left*; total RNA fractions were analyzed by small RNA Northern blotting with a *ChlI* (*SUL*) probe. Total protein fractions were analyzed by Western blotting developed with *ChlI* antibodies. *D, bottom*, WT, *era1–2* and *plp-3* plants expressing the SUC:SUL (SS) hairpin. *Top*, *ChlI* (*SUL*) siRNA levels in leaves from pools of 5 plants from the F3 generation; total RNA fractions were analyzed by small RNA Northern blotting with a *ChlI* (*SUL*) probe.

Because isoprenoid biosynthesis is required for miRNA and siRNA activity in *Arabidopsis* (21), we tested whether protein farnesylation could also play a role in small RNA function. We first introduced reporter systems for miR156 (41), miR171 (42), and miR403 activity into *era1–2* and analyzed reporter expression or activity in WT compared with *era1–2*. We also monitored mRNA accumulation of a number of endogenous miRNA targets in *era1* and *plp* mutants. These tests did not reveal clear defects in miRNA function (Fig. S1). In several cases, however, mutation of *bona fide* miRNA pathway components does not lead to observable defects in miRNA function on their own, but create a sensitized background in which defects become clearly observable only when combined with other weak mutations in miRNA pathway factors. For example, mutants in the *Arabidopsis* HSP90 co-chaperone SQN show weak miRNA-related defects on their own, but the importance of SQN for miRNA activity is revealed by its spectacular genetic interaction with weak *ago1* mutant alleles (8). Similarly, at low temperature, mutants in the *C. elegans* AGO protein ALG-1 show weakly penetrant defects in developmental transitions controlled by the *lin-4* and *let-7* miRNAs, but those phenotypes become strongly exacerbated upon mutation of components of the Golgi-associated retrograde protein complex that affects miRNA levels, including those of the *let-7* family (43). To test for such synthetic interactions, we constructed two sets of double mutants with *era1–2*: the first with a hypomorphic mutant allele of the key miRNA biogenesis factor *DICER-LIKE1* (*dcl1–11* (44, 45)), and the second with the hypomorphic *ago1–27* allele (46). In contrast to *dcl1–11* and *era1–2* single mutants, *dcl1–11/era1–2* double mutants formed cup-shaped cotyledons, filament-like structures instead of flowers and trumpet-shaped leaves (Fig. 1*A*), reminiscent of mutants in the miR165/miR166-binding site of the target REVOLUTA (REV) that encodes a transcription factor (47). Some direct REV targets showed stronger up-regulation in *era1–2/dcl1–11* double mutants than in either single mutant, but this trend was not general to all target genes of transcription factors repressed by miRNAs (Fig. 1*B*). A genetic interaction with *ago1–27* was also detected, because *ago1–27/era1–2* double mutants were clearly smaller than *ago1–27* single mutants, and were completely sterile in contrast to either single mutant (Fig. S2). Although these clear genetic interactions do not allow precise molecular conclusions on links between protein farnesylation and miRNA action to be drawn, they do support the implication of protein farnesylation in developmental functions linked to, or possibly controlled by, the miRNA pathway.

### Farnesyl transferase mutants have weak defects in noncell autonomous siRNA activity

We next introduced the *era1–2* mutation into the SUC:SUL silencing system that uses a phloem-specific hairpin construct to produce noncell autonomously acting siRNAs to silence the magnesium chelatase subunit ChlI (SUL). Such vein-centered ChlI silencing gives rise to a yellow-striped leaf phenotype in WT (48). SUL siRNAs are generated by a DICER-LIKE4 (DCL4)-dependent pathway different from the DCL1-dependent miRNA biogenesis pathway, but both pathways implicate the same downstream silencing effector AGO1 (49–51). We observed reduced SUL silencing in *era1–2* (Fig. 1*C*). The reduction in SUL silencing was incomplete, such that 48% of *era1–2* individuals showed suppressed silencing, whereas 52% had a silencing pattern similar to WT. *era1–2* individuals with suppressed silencing had siRNA levels similar to WT individuals with clear SUL silencing (Fig. 1*C*). These observations suggest that farnesyl transferase is required either for full activity of AGO1-dependent SUL siRNAs or for their cell-to-cell movement. Curiously, when the SUL-silencing system was introduced into the *plp-3* mutant defective in the farnesyl transferase α-subunit, we observed increased SUL silencing in older leaves, and strongly reduced SUL silencing in emerging leaves (Fig. 1*D*), perhaps supporting a defect in movement rather than silencing activity *per se*. Taken together with the strong genetic interaction between *DCL1* and *ERA1*, these data suggest the existence of functional links between protein farnesylation and gene regulation by small RNAs, and motivated us to further explore such links molecularly.

### A proteomic screen for membrane-associated AGO1 interactors

**Figure 2.**
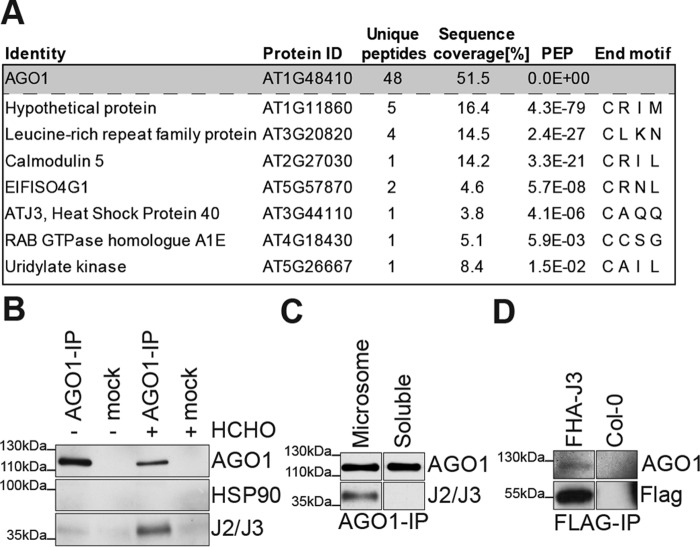
**AGO1 interacts with farnesylated J2/J3 in membrane fractions.** *A,* list of C*AAX* motif proteins identified in AGO1, but not in mock immunopurifications from deoxycholate-solubilized microsome fractions prepared from formaldehyde cross-linked 16-day-old seedling tissue. AGO1 is included to document efficiency of the purification. A list of all proteins identified is provided under the supporting Information. *B,* co-immunoprecipitation analysis of AGO1 and J2/J3 from membrane fractions. *HCHO* indicates formaldehyde cross-linking of seedling tissue prior to lysis. *Mock*, nonfunctional rabbit IgG applied in the same concentration as AGO1 antibody. *C,* co-immunoprecipitation analysis of AGO1 and HSP40 from membrane and soluble fractions. Noncross-linked tissue from 16-day-old seedlings was used. The samples were loaded on the same gel; *white* separation lines indicate the presence of additional lanes on the original gel. *D,* Western blot analysis of FLAG immunoprecipitates prepared from deoxycholate-solubilized membrane fractions obtained from an FHA-J3 transgenic line. Noncross-linked tissue from 16-day-old seedlings was used. *Mock*, parallel FLAG immunoprecipitation from nontransgenic parental line (Col-0). The samples were loaded on the same gel; *white* separation lines indicate the presence of additional lanes on the original gel.

Because AGO1 does not harbor a C-terminal C*AAX* motif, we hypothesized that one or more AGO1-associated proteins may be farnesylated. We focused on membrane fractions to identify such putative farnesylated AGO1 interactors, because membrane association of AGO1 is affected in *hmg1* mutants (21), and because farnesylation may drive membrane association of modified proteins (52). We performed large-scale immunoaffinity purification of deoxycholate-solubilized AGO1complexes from microsome fractions of formaldehyde cross-linked seedling tissue (Fig. S3). After reversal of formaldehyde cross-links, co-purifying proteins were identified by mass spectrometry and searched for the presence of C-terminal C*AAX* sites. This approach yielded a short list of candidates in which J3, one of more than 100 HSP40 chaperones in *Arabidopsis*, was of particular interest (Fig. 2*A*). Despite detection of only a single J3 peptide, the identification of J3 in the AGO1 purification was robust (Fig. S4). J3 and its less highly expressed isoform J2 are farnesylated *in planta* (40), and may be relevant to small RNA function: the J2/J3 orthologue in *Drosophila*, Droj2, was identified as a prominent interactor of Ago1 and Ago2 (5), and was one of five chaperones required for *in vitro* reconstitution of chaperone-mediated siRNA loading of Ago2 (16). In addition, the mammalian J2/J3 orthologs in the DnaJA subfamily are also farnesylated (53), and were found as Ago2 interactors in a proteomics study of factors associating with core RNA silencing components (54). We therefore focused on J2/J3 to analyze how protein farnesylation may influence small RNA function, in particular AGO1.

### J2/J3 interact with AGO1 in membrane fractions

To confirm the association of AGO1 with J2/J3 in membrane fractions, we performed co-immunoprecipitation assays with either formaldehyde cross-linked or untreated seedling tissue. J2/J3 was readily detectable in AGO1 immunoprecipitations from microsomal fractions, but not from the same amount of AGO1 immunoprecipitated from soluble fractions (Fig. 2, *B* and *C*). We also used a stable transgenic line expressing N terminally 2×FLAG-2×haemagglutinin (FHA)-tagged J3 (40) to confirm that AGO1 was found in FLAG immunoprecipitations of deoxycholate-solubilized microsomes prepared from this line (Fig. 2*D*). Thus, J2/J3 and AGO1 interact in membrane fractions. Interaction may also occur in soluble fractions, particularly given that a sizeable part of farnesylated J2/J3 is soluble (40), but if so, it is below the detection limit of our co-immunoprecipitation assays. We note that despite higher expression levels than J2/J3, HSP90 was not detected in our AGO1 immunopurifications (Fig. 2*B*). This argues that the J2/J3 signal does not arise as a consequence of nonspecific chaperone interactions post lysis, and is consistent with the finding that interactions between HSP40 and native clients can be of higher affinity than those involving other chaperones (13).

### Farnesylation affects the interaction of J2/J3 with AGO1

**Figure 3.**
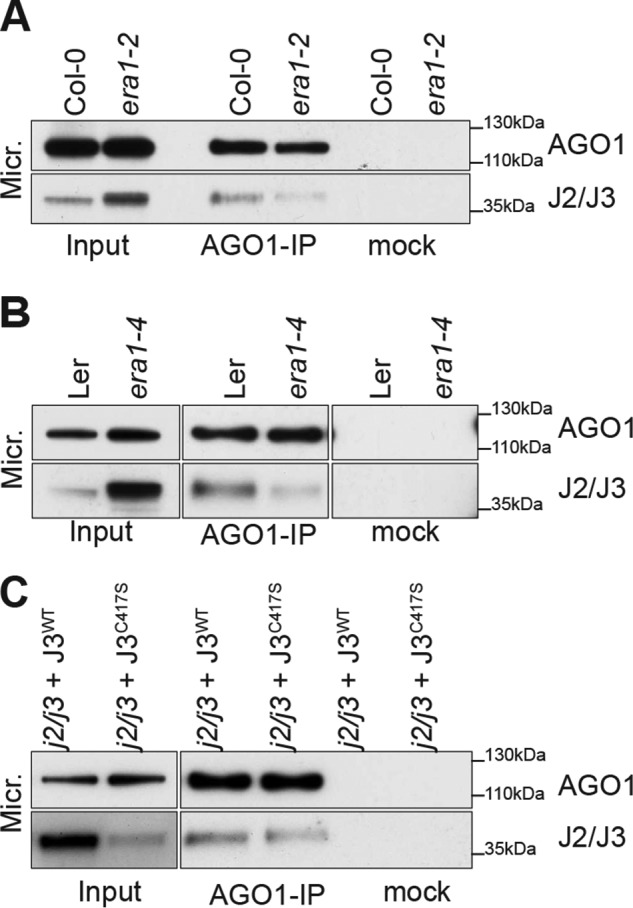
**Farnesylation of J2/J3 is required for AGO1 interaction.** *A–C,* AGO1 immunoprecipitates from deoxycholate-solubilized microsome fractions (16-day-old seedlings) analyzed by AGO1 and J2/J3 antibodies. *Inputs* were adjusted to ensure equal recovery of AGO1 in the immunoprecipitations.

HSP40 farnesylation may be necessary not only for membrane association, but also for interaction with clients, as in the case of the STE11 kinase in *Saccharomyces cerevisiae* (55). We used co-immunoprecipitation assays to test whether the association between AGO1 and HSP40 also depends on farnesylation. We used two independent mutant alleles of *ERA1* (*era1–2* in accession Col-0 and *era1–4* in accession Ler) for these analyses. In addition, we used transgenic lines expressing either J3^WT^ or the farnesylation-deficient point mutant J3^C417S^ in *j2/j3* double knockout backgrounds (40). We adjusted immunoprecipitation inputs such that equal amounts of AGO1 were immunoprecipitated from each sample. These experiments showed that substantially less J2/J3 was immunoprecipitated with AGO1 from *era1* mutants than from WT controls (Fig. 3, *A* and *B*). The same tendency, albeit less pronounced, was repeatedly observed by comparison of J2/J3 amounts immunoprecipitated with AGO1 in J3^WT^ *versus* J3^C417S^ (Fig. 3*C*). Despite the use of the endogenous J3 promoter, these lines express substantially higher levels of J3 protein than WT (40), and it is possible that the high expression levels of J3 in both J3^WT^ and J3^C417S^ transgenic lines affect the outcome of the co-immunoprecipitation assays. We conclude that farnesylation of J2/J3 plays a role in their association with AGO1 in membrane compartments, but note that the data do not rule out the possibility that additional farnesylated proteins, or indeed farnesyl transferase itself, may influence J2/J3-AGO1 interaction.

### J2/J3 and HSP90 influence membrane association of AGO1

**Figure 4.**
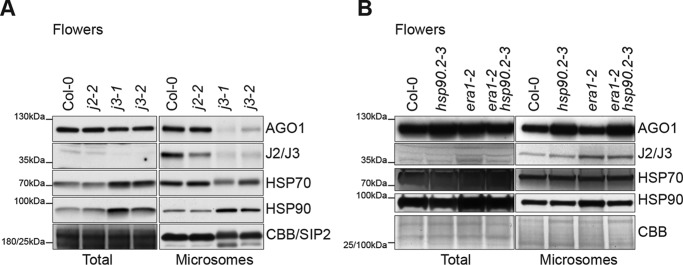
**Mutation of J3 and HSP90 chaperones affects membrane association of AGO1.** *A* and *B,* Western blotting of AGO1, J2/J3, HSP70, and HSP90 in total and microsome fractions prepared from inflorescence lysates of the indicated genotypes. For total fractions, equal loading was verified by Coomassie staining (*CBB*); for microsome fractions, Western blots were probed with SIP2 antibodies. Different sections of the membranes used for analysis of total lysates and microsome fractions were used for verification of protein loading; the *leftmost* molecular weight standard refers to the membrane used for total lysates, whereas the *rightmost* molecular weight standard refers to the membrane used for microsome fractions.

Because the J2/J3-AGO1 association was detected only in membrane fractions, and because inhibition of HSP90 leads to reduced levels of membrane-associated Ago2 in mammalian cells (20), we tested whether mutation of J2/J3 and HSP90 may influence membrane association of *Arabidopsis* AGO1. Membrane-associated AGO1 levels were appreciably lower in inflorescence tissue in *j3*, but not in *j2*, mutants (Fig. 4*A*). The same trend, although less pronounced, was observed in seedlings (Fig. S5*A*). A similar difference between analysis of inflorescences and seedlings was also observed for the ago1–38 mutant protein (Fig. S5*B*), previously shown to be less abundant specifically in membrane fractions (21). On the other hand, the levels of membrane-associated AGO1 were specifically increased in the *hsp90.2–3* mutant (Fig. 4*B*), containing a lesion in the ATPase domain encoded by one of five *HSP90* genes in *Arabidopsis* (56). These observations show that mutations in the HSP90 pathway affect membrane association of AGO1 in *Arabidopsis*, and indicate that the relationship between chaperone activity and level of membrane-associated AGO1 is not simple.

### J2/J3 farnesylation influences membrane association of AGO1

**Figure 5.**
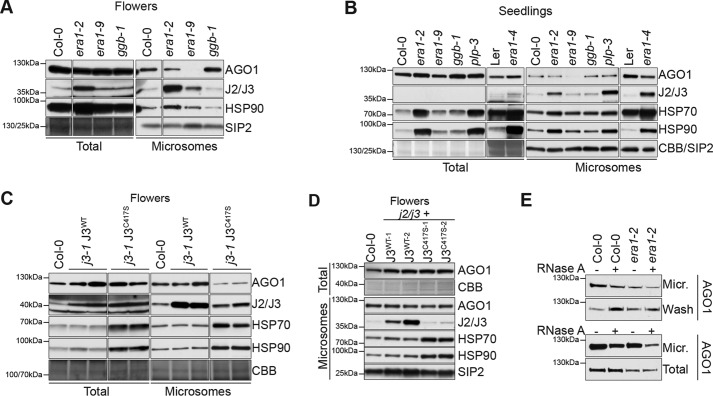
**Farnesylation of J3 affects membrane association of AGO1.** *A–D*, Western blotting of AGO1, J2/J3, HSP70, and HSP90 in total and microsome fractions prepared from inflorescence lysates of the indicated genotypes. For total fractions, equal loading was verified by Coomassie staining (*CBB*); for microsome fractions, Western blots were probed with SIP2 antibodies. In *A–C*, different sections of the membranes used for analysis of total lysates and microsome fractions were used for verification of protein loading. In these cases, the *leftmost* molecular weight standard refers to the membrane used for total lysates, whereas the *rightmost* molecular weight standard refers to the membrane used for microsome fractions. *E,* RNase sensitivity of membrane-bound AGO1. Western blotting showing AGO1 abundance in microsome fractions with or without treatment with RNase A (10 μg/ml). *Top*, RNase A was added to hypertonic lysis buffer and was present throughout microsomal fractionation. *Bottom*, microsomes were prepared with RNase-free buffer, but were resuspended and washed for 15 min in lysis buffer with or without 10 μg/ml of RNase A.

Next, we analyzed whether J2/J3 farnesylation influences membrane association of AGO1 and J2/J3. We first noted that *hmg1–4* mutants exhibited reduced levels not only of AGO1, but also of J2/J3 in membrane fractions (Fig. S5*C*) (21). In addition, membrane-bound AGO1 levels were clearly diminished in the *era1–9* intron insertion allele in flowers (Fig. 5*A*) and seedlings (Fig. 5*B*), indicating that farnesylation is required for membrane association of AGO1 (description of analysis of other *era1* and *plp* alleles follows below). In *j3-1* lines expressing J3^C417S^, levels of AGO1 and J2/J3 in membrane, but not in total fractions, were lower than in *j3-1* lines expressing J3^WT^ (Fig. 5*C*). Taken together, these data indicate that the farnesylation of J3 enhances the membrane association of AGO1.

### Confounding effects on membrane association of AGO1 upon loss of J2/J3 farnesylation coincides with chaperone induction

The analyses of membrane association of AGO1 shown in Fig. 5, *A* and *B,* reveal that *era1–9*, but not *era1–2*, *plp-3*, or *era1–4*, exhibited lower AGO1 levels in membrane fractions. *ggb* mutants also behaved like WT, as expected. Examination of J2/J3 levels showed that J2/J3 protein (Fig. 5, *A* and *B*) and mRNA (Fig. S6) were strongly induced in *era1–2*, *plp-3*, and *era1–4*. HSP70 and HSP90 were also induced in these mutants (Fig. 5, *A* and *B*) (40). Given the complex relationship between HSP40/HSP70/HSP90 pathway activity and membrane-association of AGO1 revealed by the analysis of *j3* and *hsp90.2* single mutants, these strong chaperone inductions complicate the interpretation of the results, because opposing effects on AGO1 membrane association are likely to be taken into account. The same concern applies to the analysis of *j2*/*j3* double knockout mutants expressing J3^WT^ or J3^C417S^ that showed little effect on AGO1 membrane association upon mutation of the J3 farnesylation site (Fig. 5*D*). In these lines, J3 was overexpressed despite the use of the endogenous J3 promoter, and HSP70 and HSP90 were strongly induced in J3^C417S^ lines (40) (Fig. 5*D*). We considered the possibility that both RNA-dependent and RNA-independent pools of membrane-associated AGO1 may exist and may have different requirements for J2/J3 farnesylation. Experiments using RNase A treatment during microsome fractionation showed that a sizeable fraction of membrane-associated AGO1 is RNA-dependent, but revealed no major difference in RNase A sensitivity between WT and *era1–2* (Fig. 5*E*). We also tested the effect of J2/J3 knockdown in *era1–2* by introducing a dexamethasone-inducible artificial miRNA targeting J2/J3 (40). Knockdown of J2/J3 in *era1–2* did not lead to appreciable changes in levels of membrane-associated AGO1 (Fig. S7, *A–C*), indicating that induction of J2/J3 alone does not explain the high levels of AGO1 in membrane fractions in *era1–2* mutants. We conclude from the analyses of membrane-associated AGO1 in chaperone and farnesyl transferase mutants that J2/J3 farnesylation influences membrane association of AGO1, consistent with the enhancement of AGO1-J2/J3 association by farnesylation. The data also show, however, that simple lack of J2/J3 farnesylation is not sufficient to lose AGO1 from membrane fractions, possibly as a consequence of chaperone induction in mutants with strong defects in J2/J3 farnesylation.

### J2/J3 farnesylation is not required for RER localization of AGO1

**Figure 6.**
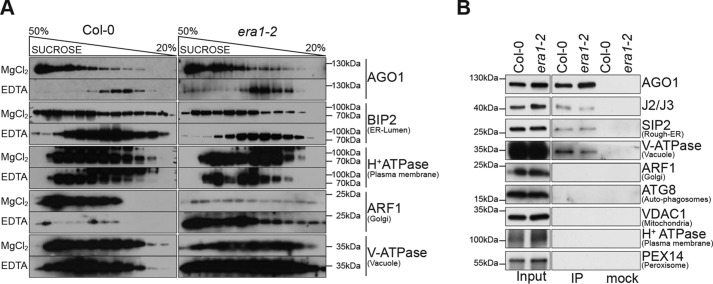
**Farnesylation of J2/J3 does not affect AGO1 localization.** *A,* analysis of microsome fractions obtained from 12-day-old liquid-culture grown seedlings by 20–50% sucrose gradient centrifugation. Aliquots of sucrose gradient fractions were analyzed by Western blotting with antibodies against the indicated proteins. MgCl_2_, microsomes resuspended in buffer containing 5 mm MgCl_2_; EDTA, microsomes resuspended in buffer containing 2 mm EDTA. *B,* AGO1 membrane IPs were prepared in the absence of detergent. Immunoprecipitates were analyzed by Western blots with the indicated antibodies. Microsomes resuspended in buffer without detergent were used as inputs.

Because AGO1 associates with membrane fractions in *era1–2* and J3^C417S^ mutants, yet interacts less well with J2/J3 in these backgrounds, we considered the possibility that J2/J3 farnesylation would be required for AGO1 localization to the correct endomembranes, probably the RER (25). To test this, we performed sucrose gradient centrifugation of microsomal fractions from Col-0 and *era1–2* in the presence and absence of Mg^II^ ions. Chelation of Mg^II^ dissociates ribosomes from the RER, producing a characteristic density shift of RER membranes, unlike membranes derived from other compartments (57). Nearly all of the AGO1 signal shifted from heavy to light fractions upon chelation of Mg^II^ by EDTA in both WT and *era1–2*, similar to the RER marker BiP (Fig. 6*A*). This confirms AGO1 localization to the RER in WT, and indicates that RER localization of AGO1 is not abrogated upon loss of farnesyl transferase. This conclusion was confirmed by AGO1 immunoprecipitation from microsomes resuspended in buffer devoid of detergent, such that entire membrane pieces were immunoprecipitated with AGO1 (“membrane IPs”) (26). Immunoprecipitated fractions contained markers for the RER and for the vacuole, but not for other membrane compartments. Importantly, the amount of RER and vacuole markers detected in membrane IPs from Col-0 and *era1–2* was nearly identical (Fig. 6*B*). Thus, AGO1 is associated with the RER in *era1–2*. We conclude that lack of farnesyl transferase, and hence of J2/J3 farnesylation, does not lead to gross mislocalization of membrane-bound AGO1.

### Membrane-bound AGO1 is loaded in the absence of J2/J3 farnesylation

**Figure 7.**
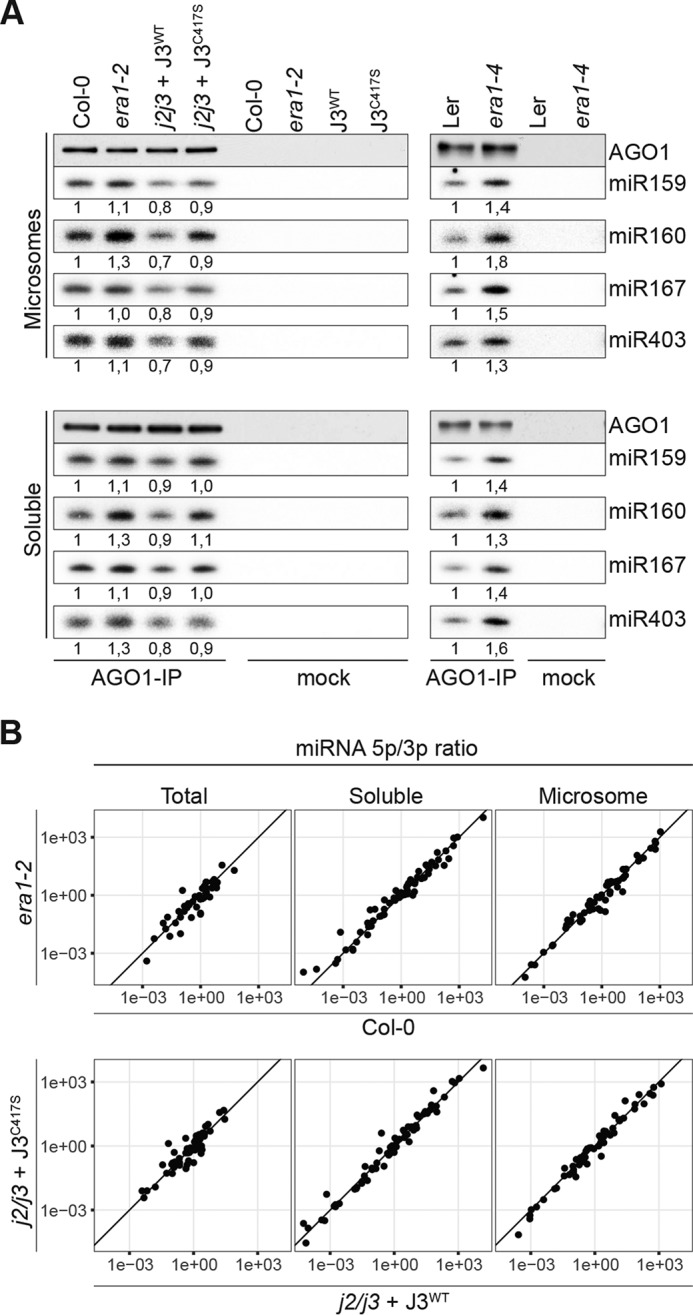
**Membrane-bound RISC is loaded in the absence of J2/J3 farnesylation.** *A,* AGO1 immunoprecipitates from soluble and membrane fractions analyzed by small RNA Northern blots. An aliquot of the immunoprecipitate was used for AGO1 Western blots to document equal recovery of the AGO1 protein from the different samples. The same Northern membrane was used for consecutive hybridizations to all probes. *B,* small RNA-seq analysis of small RNA isolated from AGO1 immunoprecipitates from microsome fractions. Ratios of read counts of pairs of miRNA/miRNA* are depicted for the indicated genotypes.

Because the HSP90 pathway is necessary for RISC loading, we tested the possibility that J2/J3 farnesylation is required for RISC loading specifically in membrane compartments. Small RNA populations in total microsome and in microsomal AGO1-bound fractions were analyzed by Northern blotting, again normalizing immunoprecipitation inputs to AGO1 protein quantity. These analyses showed that AGO1 isolated from *era1–2* or from the transgenic *j2/j3 J3^C417S^* line contained similar, or perhaps slightly increased, levels of miRNAs compared with their corresponding WT control (Fig. 7*A*). We also tested the *era1–4* mutant in accession of Ler, and observed small, but consistent increases in levels of miRNAs bound to AGO1 (Fig. 7*A*), perhaps as a consequence of the very strong chaperone induction in this mutant background (Fig. 5*B*). The AGO1-miRNA complexes analyzed from *era1–2* and J3^C417S^ represented mature, loaded RISC, because deep sequencing of AGO1-small RNA complexes immunopurified from membrane fractions showed that miRNA/miRNA* ratios were similar between Col-0 and *era1–2,* and between J3^WT^ and J3^C417S^ (Fig. 7*B*). Thus, loss of J2/J3 farnesylation does not impair small RNA loading into AGO1 in membrane compartments.

### J2/J3 farnesylation influences the distribution of miRNA between polysome-bound and -unbound fractions

**Figure 8.**
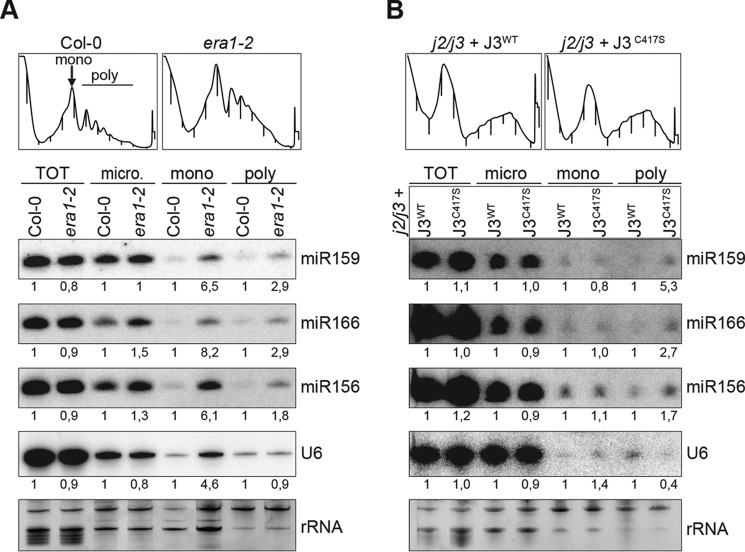
**Increased miRNA association with membrane-bound polysomes in *era1* and *j2/j3*+*J3^C417S^*.** *A* and *B,* top, absorbance measured at 260 nm as a function of fraction number from a sucrose gradient used to separate polysome- and monosome-containing fractions from lighter fractions of microsome pellets isolated from the indicated mutant or transgenic lines. *Bottom*, small RNA Northern blot analysis of miRNA levels in total RNA (*TOT*) and RNA extracted from total microsomal (*micro*), monosome-containing (mono) and polysome-containing (*poly*) microsomal fractions. Several polysome-containing fractions were pooled as indicated on the *top left panel* in *A*.

In mammalian cells grown to confluency, miRNA association with polysomes is increased, and, possibly as a consequence thereof, their activity is reduced (58). We therefore asked whether J2/J3 farnesylation might influence the distribution of miRNAs in polysome-bound *versus* lighter fractions. We focused on membrane fractions, and used sucrose gradient centrifugation to prepare polysomes from microsomal fractions of Col-0, *era1–2*, *j2–2*/*j3-2* + J3^WT^, and *j2–2*/*j3-2* + J3^C417S^. miRNA levels were determined in the total microsomal fraction, the monosomal fraction, and in pooled polysomal fractions. These analyses revealed that miRNAs were clearly enriched in polysomal fractions in *era1–2* mutants compared with WT (Fig. 8*A*). A clear enrichment, although less pronounced, was also observed in the *j2/j3* + J3^C417S^ transgenic line (Fig. 8*B*). We conclude that loss of J2/J3 farnesylation shifts membrane-associated miRNAs toward the polysome-bound fraction.

## Discussion

### Are AGO proteins conserved clients of farnesylated HSP40?

Our study identifies the farnesylated HSP40 chaperones J2 and J3 as interactors of AGO1 in membrane fractions, and provides evidence for the relevance of protein farnesylation, and of J2/J3 farnesylation in particular, for membrane association of AGO1 and for the association of small RNAs with polysomes. The implication of HSP40 farnesylation in small RNA function is likely to be broadly conserved. First, a study of genes co-evolving with RNA-silencing genes across 83 eukaryotic genomes identified farnesyl transferase α-subunit (FNTA-1) as a top hit, and the *C. elegans* homologue FNTA-1 was validated experimentally as relevant for RNA silencing (59). Second, proteomics studies in *Drosophila* and human cells have shown that J2/J3 orthologues associate with AGO proteins in both organisms (5, 54), suggesting that they could play roles in small RNA function similar to what we have described here in *Arabidopsis*. We note that the potential existence of conserved links of HSP40 farnesylation to RNA silencing is of considerable biomedical importance, because several drugs, including the widely used statins, inhibit protein farnesylation in humans (60).

### Relationships between the HSP40/70/90 chaperone pathway and levels of membrane-associated AGO1

**Figure 9.**
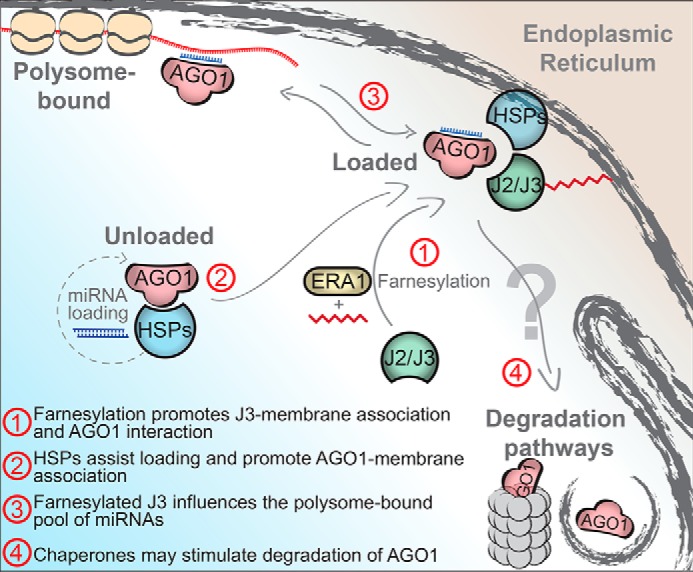
**Tentative model summarizing the results on J2/J3 farnesylation and membrane-bound AGO1.** The model puts forward the idea that the HSP40/70/90 pathway, and in particular farnesylated J2/J3, may engage in multiple functional interactions with membrane-bound AGO1. These may include promotion of membrane association of J3 (40) and AGO1 interaction (this work), AGO1 loading (6), the amount of polysome-bound RISC (this work), and, perhaps, a role in regulated proteolysis (not directly supported by evidence, but consistent with observations reported here and on roles of chaperones in assisting proteolysis of other client proteins).

Given the central function of membrane-bound AGO proteins for RNA silencing, the mechanisms underlying their endomembrane recruitment and turnover constitute a highly important, yet unresolved topic. Our study provides new insight into this process, tentatively summarized in Fig. 9: we show that J3 farnesylation is required for normal steady-state levels of membrane-bound AGO1, and that perturbation of the HSP40/HSP70/HSP90 system may have different effects of those levels. Early studies in human cells showed that inhibition of HSP90 activity led to loss of Ago2 from Golgi membranes (20), but given that HSP90 activity is mandatory for Ago2 loading and that unloaded Ago2 is turned over rapidly (7, 28), these results may simply reflect accelerated degradation of Ago2. We show that farnesylation of J2/J3 is not required for loading of AGO1 in *Arabidopsis*. Nonetheless, it remains unclear whether the requirement for farnesylated J2/J3 reflects a function in actual recruitment of AGO1, or in preventing degradation of membrane-bound AGO1. As put forward in Fig. 9, it is possible that the opposing effects observed on steady-state levels of AGO1 in membrane fractions in *j3* and *hsp90.2–3* mutants reflect multiple functional interactions between the HSP40/HSP70/HSP90 system and AGO1, for example, in loading, membrane recruitment, and regulated turnover, by the proteasome and/or autophagy (27, 61). Such opposing roles of the HSP90 system have been observed on a well-studied class of plant HSP90 clients, the cytosolic immune receptors known as Resistance (R) proteins. R proteins require HSP70/HSP90 for activation, presumably due to assisted conformational changes, but HSP90 and the co-chaperone SGT1b are also required for chaperone-assisted proteolysis of many R proteins (62, 63). A similar dual involvement of the HSP40/70/90 chaperone system in recruiting and maintaining AGO1 levels at membranes could potentially explain the somewhat contradictory results obtained here with mutants in different elements of the chaperone system. This model of multiple functional interactions between the chaperone machinery and AGO1 (Fig. 9) may also explain the confusing observation that complete loss of J2/J3 farnesylation has a different, and less clear, effect on membrane association of AGO1 than loss of J3 farnesylation alone. If, for instance, loading, membrane recruitment, and proteolysis have different sensitivities to chaperone dosage, the strong induction of HSP40/70/90 observed upon complete loss of J2/J3 farnesylation could underlie the different results observed on membrane association of AGO1 between the *j3*+*J3^C417S^* and *j2/j3*+*J3^C417S^* transgenic lines, and even between *era1* mutants carrying either deletion (*era1–2*) or intron insertion (*era1–9*) alleles.

### Effect of HSP40 farnesylation on RISC activity

Our search for involvement of protein farnesylation in RISC activity revealed two effects. First, noncell autonomously acting hairpin-derived siRNAs gave rise to reduced silencing in *era1* mutants. It is likely that these results do not reflect a defect in silencing activity of RISC, but rather a defect in small RNA movement, because *plp* mutants showed increased silencing in older leaves (photosynthetic sources), but reduced silencing in emerging leaves (photosynthetic sinks) in which the SUC2 promoter is not active (64). Second, *era1* interacted very strongly genetically with the hypomorphic *dcl1* mutant *dcl1–11*, and double mutants displayed developmental defects consistent with defective miRNA activity. Such strong genetic interaction is often observed between mutations that each have weak effects on the same pathway. Our observation that miRNAs are shifted to the polysome fraction in *era1* and in farnesylation-deficient J2/J3 mutants (Fig. 9) may constitute a molecular explanation for this genetic observation. If, as in mammalian cells (58), increased polysome association of miRNAs reduces their activity, the effect may become readily observable only in genetic backgrounds with decreased total levels of miRNAs, such as *dcl1–11*. If so, it will be of key importance to determine the molecular nature of these inactivated polysome-associated RISCs, and to understand how farnesylated HSP40 is involved in preventing their accumulation.

## Experimental procedures

### Plant growth conditions

Seeds were sterilized in 96% ethanol for 5 min, then in 1% NaOCl solution (AppliChem) for 5 min and rinsed three times in sterile water before being sown on Murashige-Skoog (MS) agar plates (4.3 g/liter of MS salts, 0.8% agar, 1% sucrose) or in soil containing 4% perlite and 4% vermiculite. For seedling analyses, plants were grown for 16 days on MS at constant temperature (21 °C) and a 16-h light (120 μmol m^−2^ s^−1^)/8-h darkness cycle. For analysis of inflorescences, plants were grown in growth chambers (Percival) with a 16-h photoperiod (150 μmol m^−2^ s^−1^), 21/16 °C day-night temperatures, and 70% relative humidity. For dexamethasone induction of amiR-J3, seeds were germinated on MS medium containing 10 μm dexamethasone (Sigma).

### DNA constructs and transgenic lines

The *UBQ:LUC-AGO2:3′-UTR* (UL403) reporter was constructed by USER cloning. PCR fragments of the *UBQ10* promoter, firefly luciferase (*LUC*), and *AGO2–3′-UTR* were amplified from genomic DNA or from a RD29A-LUC-NOS vector (65) with primers 70/71, 72/73, 74/77 (UL403), or with primers 70/71, 72/73, 74/75, 76/77 (Ul403m), and cloned into a derivative of pCAMBIA3300 containing a USER cassette (66). Sequences of all oligonucleotides used for cloning are shown in Table S1.

### Transformation of Arabidopsis

Plants were transformed by floral dipping with *Agrobacterium tumefaciens* strain GV3101 (67).

### Mutant genotyping and double mutant construction

*Arabidopsis* T-DNA insertion mutants were genotyped using PCR to confirm the T-DNA insertion sites and select homozygous mutants. Genotyping primers are listed in Table S1. The deletion in *era1–2* was confirmed by PCR with primers inside the *ERA1* gene body, and by the total absence of signal in quantitative RT-PCRs from RNA prepared from *era1–2* (Table S1). *era1–2/era1–2, hsp90.2–3*/+ individuals were identified by PCR in F2 populations of *era1–2* crossed to *hsp90.2–3*, using absence of product with *ERA1* gene body primers for *era1–2* genotyping, and primers 16 and 17 (Table S1) followed by AseI digestion for *hsp90.2–3* genotyping. Double homozygous plants were then identified and characterized in the F3 generation.

For construction of *era1–2/*SUC:SUL and *plp-3/*SUC:SUL, F2 populations of crosses to the SUC:SUL line (50) were subjected to BASTA selection to select for the SUC:SUL transgene. Homozygous mutants were selected by PCR as described above, and occurred at an expected frequency of roughly 25%. Seed aliquots of different F3 families were tested for homozygosity for BASTA resistance. BASTA homozygous families were used for phenotypic and molecular analyses that were performed on leaves from 4–5-week-old soil-grown plants. A list of mutants used and generated in the study is compiled in Table S2.

### Isolation of RNA

RNA was extracted from ∼100 mg of ground tissue using 1 ml of TRIzol (TRI Reagent, Sigma) according to the manufacturer's instructions. RNA concentration was measured using a nanodrop spectrophotometer (ND-1000, Fisher Scientific). RNA quality was visualized by gel electrophoresis on 1% agarose and ethidium bromide gel staining. Small RNA from immunopurified AGO1 was extracted by TRIzol as above, but was precipitated from the aqueous phase after chloroform extraction in the presence of 10 μg of glycogen as a carrier.

### Northern blotting

5–20 μg of purified total RNA was mixed with 4× loading buffer (20 mm HEPES, 1 mm EDTA, 50% formamide, 3% glycerol, bromphenol blue, pH 7.8) and heated at 95 °C for 2 min before being snap frozen on ice and loaded on pre-heated 18% acrylamide (19:1, Serva), Tris borate-EDTA (TBE) gels containing 6 m urea. Gels were run at 90 V for ∼3 h and then blotted by wet transfer to an Amersham Biosciences Hybond-NX (GE Healthcare) membrane for 1 h at 80 V in a Mini Trans-blot cell (Bio-Rad) on ice. RNA was chemically cross-linked to the membrane with EDC at 60 °C for 1.5 h following the procedure as described in Ref. 68. miRNA-specific probes were produced by PNK-labeling of complementary DNA oligonucleotides (T4 polynucleotide kinase, Fermentas) with [γ-^32^P]ATP, and were hybridized to membranes in PerfectHyb Plus Hybridization buffer (Sigma) overnight at slow rotation at 42 °C. Washed blots (3 × 20 min in 2 × SSC, 2% SDS at 42 °C) were exposed to imaging plates (BAS-MS, Fujifilm) and visualized using a laser scanner (Typhoon FLA 7000, GE Healthcare). Sequences of oligonucleotide probes are listed in Table S1.

### Quantitative RT-PCR

RNA was treated with DNase I (Fermentas), and converted to cDNA with Revert Aid reverse transcriptase (Fermentas) primed by oligo(dT) according to the manufacturer's instructions. Quantitative PCR was performed with the SYBR Green Mastermix (Fermentas) on a CFX Connect Real-Time System (Bio-Rad). Melting curve analysis of products amplified by each primer pair showed that they amplified a single PCR product. Actin2 was used as a normalization control. Primers used are listed in Table S1.

### Preparation of protein extracts and immunoblotting

Total seedling or inflorescence protein samples were extracted from 100 mg of ground tissue with NuPAGE lithium dodecyl sulfate sample buffer (Invitrogen) according to Ref. 69. Equal volumes of extract from different samples were separated on precast 4–20% Criterion gradient gels (Bio-Rad) before transfer to nitrocellulose membranes (Amersham Biosciences Protran Premium 0.45 μm, GE Healthcare) using a trans-blot blotting apparatus (Bio-Rad). Primary antibodies were detected using peroxidase-coupled goat anti-rabbit IgG (Sigma) and visualized using chemiluminescence SuperSignal West Femto Maximum Sensitivity Substrate (Thermo Scientific).

### Formaldehyde cross-linking

*Arabidopsis* seedlings were cross-linked by vacuum infiltrating whole MS plates in 1% formaldehyde solution 2 times for 7 min. Cross-linking was quenched by adding glycine to a final concentration of 125 mm and vacuum infiltrating for an additional 5 min (70). After cross-linking, MS plates with seedlings were washed three times in water before being picked, gently dried with paper towel, and frozen in liquid nitrogen.

### Microsome fractionation

Flower inflorescences or seedlings were snap frozen and ground to a fine powder. 1.2 ml of microsome buffer (50 mm MOPS, 0.5 m sorbitol, 10 mm EDTA, 1% BSA, Roche protease inhibitors version 11 (1 tablet/10 ml), pH 7.6)) was added to 0.2 g of ground tissue and vortexed thoroughly. Samples were spun at 8,000 × *g* for 10 min at 4 °C. Supernatants were transferred to new tubes and repeatedly spun at 8,000 × *g* until no pellet was visible. Supernatants (“total extracts”) were spun at 100,000 × *g* for 30 min at 4 °C. Pellets were resuspended in wash buffer (50 mm MOPS, 0.5 m sorbitol, 10 mm EDTA, Roche protease inhibitors version 11 (1 tablet/10 ml), pH 7.6) and re-pelleted by centrifugation at 100,000 × *g* for 30 min at 4 °C. Pellets were resuspended in a small volume of 1× PBS buffer and protein concentrations were measured using Bradford (Serva). Microsomes were solubilized in NuPAGE sample buffer (Invitrogen) or Laemmli sample buffer (Bio-Rad) before loading on SDS-PAGE gels.

### Immunoprecipitation from microsome fractions

Seedlings were grown on MS plates for 16–18 days. To avoid agar contamination in tissue samples, entire MS plates were snap frozen in liquid nitrogen, and tissue was harvested by breaking frozen seedling hypocotyls. If tissue had been formaldehyde cross-linked, seedlings were harvested individually into tubes cooled in liquid nitrogen. Five times volume (ml) per weight (g) of lysis buffer (50 mm HEPES/KOH, pH 7.5, 0.33 m sucrose, 5 mm MgCl_2_, 10 mm EDTA, Roche protease inhibitors version 11 (EDTA free, 1 tablet/10 ml)) was added to the ground tissue and vortexed thoroughly. For mass spectrometry analysis following immunoaffinity purification with native AGO1 antibody, 18 g of starting material was used, and 3 g was used for analysis of AGO1 immunoprecipitates by Western blotting. Samples were spun at 8,000 × *g* for 10 min at 4 °C and filtered through a layer of miracloth (Calbiochem) to remove crude nonsolubilized debris. Supernatants were transferred to new tubes and repeatedly spun at 8,000 × *g* until no pellet was visible. The crude extract (Total fraction) was further spun in an ultracentrifuge at 100,000 × *g* for 1 h at 4 °C. Supernatants were discarded or used for immunoprecipitation of soluble fractions after addition of NaCl to a final concentration of 150 mm and Nonidet P-40 to a final concentration of 0.5%. Pellets were resuspended in Resuspension buffer (20 mm HEPES/KOH pH 7.5, 0.33 m sucrose, 5 mm MgCl_2_, Roche protease inhibitors version 11 (EDTA free, 1 tablet/10 ml)) and re-pelleted by centrifugation at 100,000 × *g* for 30 min. Pellets were resuspended in a small volume of PBS buffer (10 mm Na_2_HPO_4_, 1.8 mm KH_2_PO_4_, 137 mm NaCl, 2.7 mm KCl) and solubilized by adding an equal volume of 2% (w/v) deoxycholate in water. Solubilized microsomes were centrifuged at 100,000 × *g* at 4 °C to remove unsolubilized material. Supernatants were diluted 5 times with IP buffer (50 mm Tris/HCl, pH 7.5, 150 mm NaCl, 10% glycerol, 5 mm MgCl_2_, 0.1% Nonidet P-40, Roche protease inhibitors version 11 (EDTA free, 1 tablet/10 ml)). IP solutions were pre-cleaned for 30 min by rotating at 10 rpm at 4 °C with Protein A-agarose beads, then incubated overnight at 10 rpm 4 °C with 2.5 μg of AGO1 antibody (Agrisera) and either IP buffer or an IgG that does not specifically recognize any *Arabidopsis* proteins as mock. Immune complexes were incubated for 2 h under rotation at 4 °C with protein A-agarose beads and precipitated by mild centrifugation and carefully washed 4 times in cold IP buffer. AGO1 protein complexes were eluted from the beads by adding a competitive peptide (H-MVRKRRTDAPSC-NH_2_) at a final concentration of 150 μg/ml for 30 min at room temperature. Eluted AGO1 protein complexes were analyzed by mass spectrometry as detailed below or Western blotting using specific antibodies. AGO1 crude microsome IPs (membrane IPs) were performed in the same manner, but without the deoxycholate solubilization step.

The same protocol was followed for FLAG immunoprecipitation of 2×FLAG-2×HA-J3 and 2×FLAG-2×HA-J3^C417S^. In these cases, 2 g of starting material (seedlings) was used, and immunoprecipitation was done with Anti-FLAG® M2 Affinity Gel (Sigma A2220). FLAG-tagged protein was eluted from the affinity resin with 125 μg/ml of FLAG peptide (Sigma) for 30 min at room temperature.

### Sucrose gradients

Microsome fractions were isolated from seedlings grown in liquid culture for 13 days. Plants were frozen in liquid nitrogen and ground to a fine powder. 4 g of pulverized plant tissue were homogenized in 1 ml of homogenization buffer (50 mm Tris, pH 8.2, 2 mm EDTA, 20% glycerol, 1 mm DTT and protease inhibitors (Roche)) per g of tissue. In the +Mg^II^ condition, 5 mm MgCl_2_ was added to the homogenization buffer and EDTA was replaced by EGTA. The homogenate was filtered through Miracloth (Calbiochem) to remove insoluble plant debris. The filtrate was centrifuged at 5,000 × *g* for 10 min, after which the supernatant was spun for 45 min at 100,000 × *g*. The microsome pellet was washed in homogenization buffer and resuspended in 2 ml of Resuspension buffer (25 mm Tris, pH 7.5, 10% sucrose, 2 mm EDTA/EGTA, 1 mm DTT, ± 5 mm MgCl_2_, protease inhibitors) and spun again for 30 min at 100,000 × *g*. The pellet was then resuspended in 500 μl of resuspension buffer.

Sucrose density step-gradients were generated and run according to Ref. 71. Briefly, gradients were made of four different layers with different sucrose concentrations as follows: 1.25 ml of 2 m sucrose, 3.4 ml of 1.3 m sucrose, 3.4 ml of 1 m sucrose, and 2.75 ml of 0.6 m sucrose. Sucrose was dissolved in the following buffer: 10 mm Tris, pH 7.6, 2 mm EDTA/EGTA, 1 mm DTT, ±5 mm MgCl_2_, protease inhibitors. 500 μl of microsomes were loaded on top of the sucrose gradients and spun for 16 h at 100,000 × *g* at 4 °C. 13 fractions were collected and aliquots were analyzed in SDS-PAGE gels.

### Polysome fractionation

Microsome fractions were prepared from 2 g of starting material as described above. For polysome fractionation, sucrose density step gradients were used according to Ref. 72. Gradients were made of four layers with different sucrose concentrations. Each layer was obtained by mixing 1× sucrose solution (10× sucrose solution: 0.4 m Tris-HCl, pH 8.4, 0.2 m KCl, 0.1 m MgCl_2_) with 2 m sucrose. Layer 1 contained 50% sucrose, total volume 1.85 ml; layer 2: 35% sucrose, total volume 3.65 ml; layer 3: 35% sucrose, total volume 3.65 ml; and layer 4: 20% sucrose, total volume 1.35 ml. The microsome pellet was resuspended in 1.5 ml of polysome buffer (4× sucrose solution, 5 mm EGTA, 0.5% (v/v) Nonidet P-40, 300 μg/ml of heparin, 50 μg/ml of cycloheximide, 50 μg/ml of chloramphenicol, 1 unit/ml of Ribolock RNase inhibitor), loaded on top of the sucrose gradients and spun for 2 h and 45 min at 175,000 × *g* at 4 °C. 12 fractions of 1 ml each were collected from the bottom to the top concomitantly with recording of the *A*_254 nm_ profile using a spectrophotometer.

### Preparation of samples for mass spectrometry

After reducing the volume to 50 μl in a SpeedVac, samples were denatured in 6 m urea, 2 m thiourea, 10 mm HEPES, pH 8.0, in a final volume of 300 μl. Proteins were reduced with 1 mm dl-DTT for 1 h at room temperature, followed by alkylation with 5 mm 2-chloroacetamide for 1 h at room temperature. Urea concentration was brought to below 2 m by dilution with 50 mm ammonium bicarbonate and digestion was performed overnight with trypsin at a 1:20 enzyme:protein ratio. The resulting peptides were then acidified to a final concentration of 2% trifluoroacetic acid (TFA), and desalted on in-house packed C18 StageTips (73). Noncross-linked samples were denatured in 300 μl of 6 m urea, 2 m thiourea, 10 mm HEPES, pH 8.0, after which they were processed identically to cross-linked samples.

### Analysis by LC-MS/MS

Immediately prior to LC-MS/MS injection, samples were eluted from the StageTips in 40 μl of 80% acetonitrile (ACN), 0.1% formic acid (FA), and vacuum centrifuged to reduce the volume to 4 μl, after which 4 μl of 2% ACN, 1% TFA was added to each sample for acidification. Peptides were loaded onto an Easy-Spray C18, 75 μm × 50-cm column (Thermo, ES803) using 100% Buffer A (0.1% FA in water) at 720 bar, using the Thermo Easy-nLC 1000 system (Thermo Fisher Scientific, Odense, Denmark) in a single-column setup and the column oven operating at 45 °C, after which peptides were chromatographically separated using a 240-min gradient ranging from 6 to 60% buffer B (80% ACN, 0.1% FA) at a flow rate of 250 nl/min. The Thermo Scientific Orbitrap Fusion mass spectrometer (Thermo Fisher Scientific, San Jose, CA) was operated in data-dependent mode and full MS spectra were collected in the Orbitrap analyzer, scanning from 350 to 2000 *m*/*z* at a resolution of 120,000 using an automatic gain control setting of 4e5 ions or maximum injection time of 20 ms. MS^2^ spectra were obtained by isolation in the quadrupole with a 1.6 *m*/*z* window, and acquired by rapid scan analysis in the ion trap with an automatic gain control target value of 1e4 and maximum injection time of 80 ms (100 ms for cross-linked samples) for all fragmentation methods. Precursors with charge states +2 or higher were retained, and were selected in Top Speed mode for decision tree-based ion trap HCD (normalized collision energy of 35%) or ETD fragmentation. Dynamic exclusion was set to 45 s (60 s for cross-linked samples).

### Analysis of mass spectrometry data

Raw files were processed using MaxQuant (version 1.5.0.30) (74) and searched against the complete protein database from The *Arabidopsis* Information Resource (TAIR). Tryptic peptides with up to two missed cleavages were permitted, methionine oxidation, N-terminal acetylation, and STY-phosphorylation were selected as variable modifications, and cysteine carbamidomethylation as a fixed modification. Minimum peptide length was set to 6. Peptide, site, and protein FDR were all kept at 1%.

### Construction of libraries for small RNA-Seq

Libraries for Illumina sequencing were prepared from 1 μg of total seedling RNA. All libraries were generated using the NEBNext Small RNA Library Prep Set (Multiplex) (New England Biolabs) following New England Biolab instructions. The quality of purified DNA was confirmed using an Agilent Bioanalyzer and sequenced on an Illumina platform (Aros, Denmark).

### Analysis of small RNA-Seq data

Raw illumina sequencing reads were trimmed to remove adapter sequence (AGATCGGAAGAGCACACGTCTGAACTCC) using Cutadapt (75). Trimmed reads were aligned with Bowtie 2 (76) against either the *Arabidopsis thaliana* genome sequence TAIR10.26, or against the sequences of the 427 *A. thaliana* mature miRNAs annotated in miRBase v21. Reads mapped to mature miRNA were aligned using strand-specific alignment with Bowtie 2. Reads counts were calculated using samtools idxstats (for reads mapped to mature miRNA) or bedtools multicov (for reads mapped to the genome overlapping annotated features). Read counts were normalized to the total reads mapped to the genome or to the total reads mapped to miRNA, as specified under “Results.”

### Antibodies

Rabbit antibodies against HSP40 (J2/J3) have been described (40). *Arabidopsis* SUL and GFP antibodies were as described in Ref. 77. All other antibodies used in this study are commercially available and are listed in Table S3.

### Accession numbers

Small RNA-Seq data have been deposited in the European Nucleotide Archive under accession number E-MTAB-3736. Mass spectrometry raw files have been deposited to the ProteomeXchange Consortium via the PRIDE partner repository with dataset identifier PXD010197.

## Author contributions

L. S., M. F., A. B., F. V., R. L., and P. B. formal analysis; L. S. validation; L. S., M. F., A. B., F. V., and R. L. investigation; L. S., M. F., A. B., F. V., R. L., and P. B. methodology; L. S., M. F., A. B., F. V., R. L., and P. B. writing-review and editing; M. F., A. B., F. V., R. L., and P. B. data curation; M. F., A. B., F. V., and R. L. visualization; F. V., R. L., and P. B. supervision; P. B. conceptualization; P. B. funding acquisition; P. B. writing-original draft; P. B. project administration.

## Supplementary Material

Supporting Information

## Footnotes

4 The abbreviations used are:

miRNA: microRNA
RISC: RNA-induced silencing complex
AGO: ARGONAUTE
HSP: heat shock protein
RER: rough endoplasmic reticulum
C*AAX*: Cys-aliphatic-aliphatic-Xaa
ERA1: ENHANCED-RESPONSE-TO-ABSCISIC-ACID1
REV: REVOLUTA
DCL: DICER-LIKE
SUL: subunit ChlI (SULFUR)
IP: immunoprecipitation
R protein: Resistance protein
MS plates: Murashige-Skoog plates
EDC: 1-ethyl-3-(3-dimethylaminopropyl)carbodiimide hydrochloride
ACN: acetonitrile
FA: formic acid.
