# Supplementary material for: Farnesylated heat shock protein 40 is a component of membrane-bound RISC in Arabidopsis
Source: J Biol Chem. 2018 Sep 7;293(43):16608–22. doi: 10.1074/jbc.RA118.003887 (PMC6204899; doi:10.1074/jbc.RA118.003887)
Supplement: Supporting Information [file supp_293_43_16608__index.html]

Farnesylated heat shock protein 40 is a component of membrane-bound RISC in Arabidopsis — HSP40 farnesylation in membrane-bound RISC function — Supporting Information 

# Farnesylated heat shock protein 40 is a component of membrane-bound RISC in *Arabidopsis*

## Supporting Information

- Supporting Figures and Tables - Supporting Figures S1-S7 Supporting Tables S1-S3
- Protein Groups AGO1 IP - Full list of proteins identified by mass spectrometry in AGO1 IP
- Protein Groups Mock IP - Full list of proteins identified by mass spectrometry in mock IP
