# Supplementary material for: Farnesylated heat shock protein 40 is a component of membrane-bound RISC in Arabidopsis
Source: J Biol Chem. 2018 Sep 7;293(43):16608–22. doi: 10.1074/jbc.RA118.003887 (PMC6204899; doi:10.1074/jbc.RA118.003887)
Supplement: Supporting Information [file supp_RA118.003887_138017_1_supp_168974_p6n662.pdf]

SUPPORTING INFORMATION FOR THE MANUSCRIPT ENTITLED

Farnesylated Heat Shock Protein 40 is a component of membrane-bound RISC in Arabidopsis

BY

Lars Sjögren, Maïna Floris, Andrea Barghetti, Franziska Völlmy, Rune Linding and Peter Brodersen

LIST OF MATERIAL INCLUDED

Figures S1-S7

Tables S1-S3

Excel files containing the full list of protein groups identified by mass spectrometry analysis of AGO1 and mock immuno-affinity purifications of solubilized membrane fractions from formaldehyde-crosslinked seedling tissue.

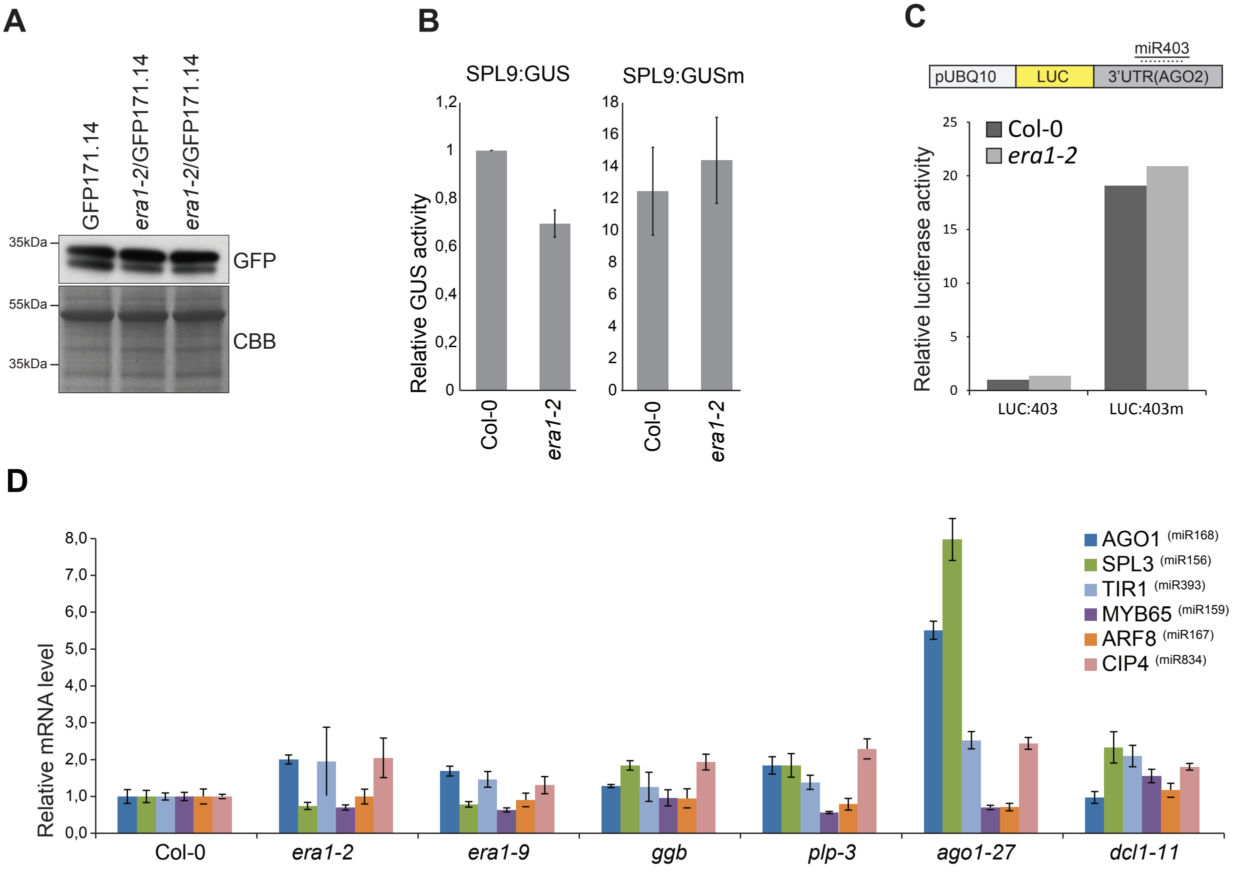

Figure S1 Defects in miRNA-guided silencing are not detectable in *era1* and *plp* single mutants

(A) *era1* mutation does not cause derepression of the stable, transgenic GFP171.14 silencing system that uses a constitutively expressed *GFP* transgene containing a miR171 target site in the 3'-UTR immediately downstream of the stop codon (1,2). Total protein fractions in 16-day old seedlings were analyzed by western blot and were developed with a GFP antibody.

(B) *era1* mutation does not cause derepression of the stable transgenic SPL9:GUS silencing system that uses a translational fusion of the natural miR156 target, SPL9 to GUS (3). The panel shows relative GUS activity in total protein extracts of Col-0 and *era1-2* 10-day-old seedlings expressing the SPL9:GUS fusion reporter (SPL9:GUS) or its control in which the miR156 target site is mutated (SPL9:GUSm). Error bars represent standard deviation between duplicates.

(C) Relative luciferase activity in total lysates of Col-0 and *era1-2* 16-day old seedlings expressing a miR403 luciferase reporter construct (LUC:403), or its control in which the miR403 target site is mutated (LUC:403m). The luciferase coding sequence is followed by the AGO2 3'UTR carrying the miR403 target site, and expressed under the control of the ubiquitin10 promoter, as shown in the, schematic representation of the construct.

(D) Quantitative RT-PCR analysis of mRNA abundance of miRNA targets in 16-day old seedlings. Error bars represent standard deviation between triplicates.

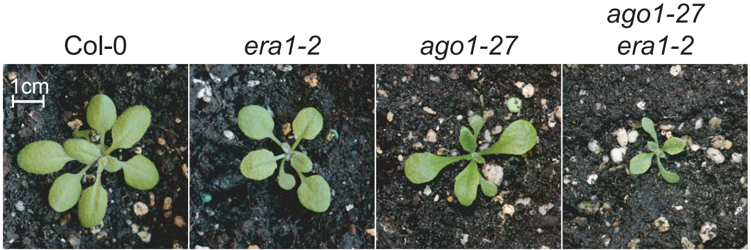

Figure S2 Genetic interaction between *ago1-27* and *era1-2*

Phenotypes of 22-day old plants of Col-0 wildtype and *era1-2*, *ago1-27*, and *era1-2/ago1-27* mutants.

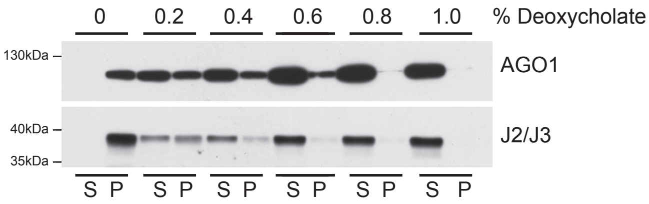

Figure S3 AGO1 protein extraction from deoxycholate-solubilized microsomes

Test of efficiency of solubilization of AGO1 and J2/J3 from microsomes by increasing concentrations of deoxycholate. Microsome pellets were resuspended in hypertonic lysis buffer containing the indicated concentrations of deoxycholate, and insoluble material was pelleted by centrifugation at 100.000x*g* for 30 minutes. S, supernatant, P, pellet.

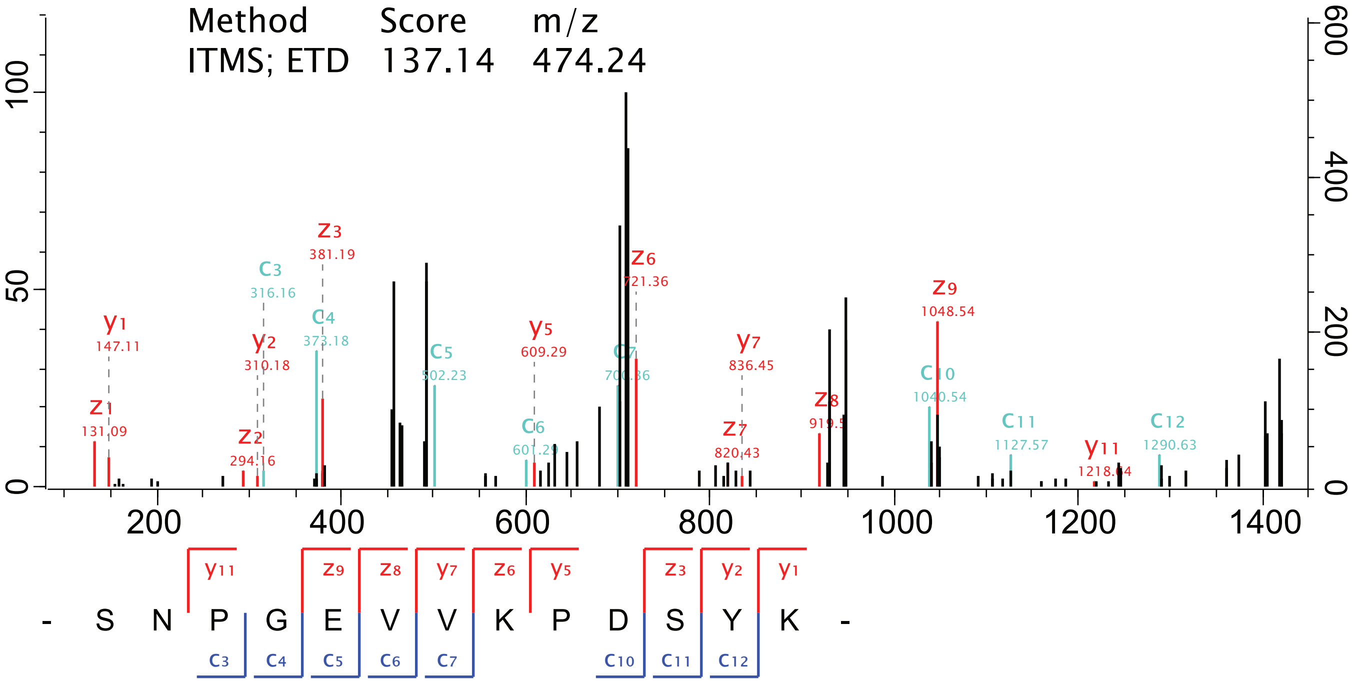

Figure S4 Identification of J3 in AGO1 immuno-purified from microsome fractions

Fusion Orbitrap MS/MS mass spectrum of the tryptic peptide of J3: SNPGEVVKPDSYK. Electron transmission-dissociation fragmentation resulted in high c-, z- and y- ion coverage of the peptide, identified with an Andromeda score of 137.14. The sample was prepared by immune-purification of AGO1 from deoxycholate-solubilized microsomes prepared from formaldehyde-crosslinked seedling tissue.

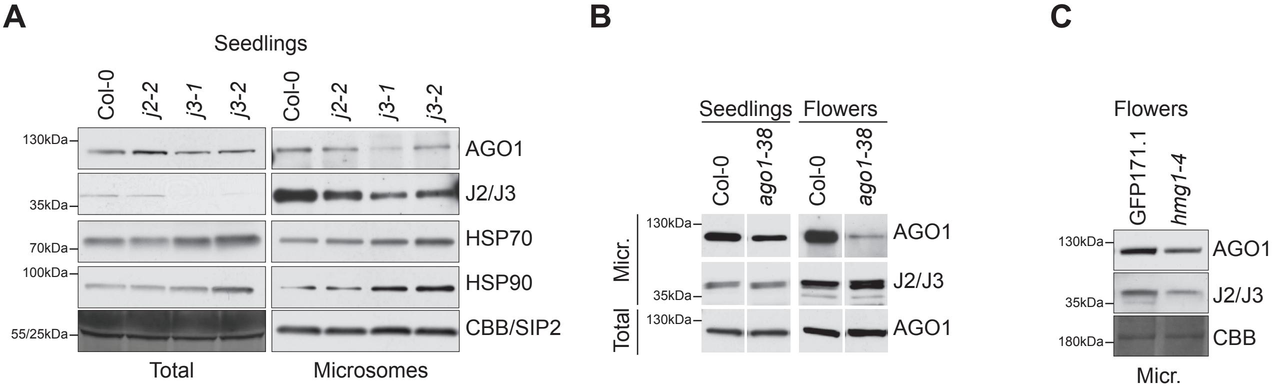

Figure S5 Effects of *j2*, *j3*, *ago1-38* and *hmg1-4* single mutations on membrane association of AGO1

(A-C) Western blot of AGO1, J2/J3, HSP70, and HSP90 in total and microsome fractions prepared from lysates of the indicated genotypes. For total fractions, equal loading was verified by Coomassie staining (CBB); for microsome fractions, western blots were probed with SIP2 antibodies. In panel A, the loading controls panels come from different sections of the full membranes: the leftmost molecular weight marker corresponds to the membrane with total lysates, the rightmost molecular weight marker corresponds to the membrane with microsome fractions.

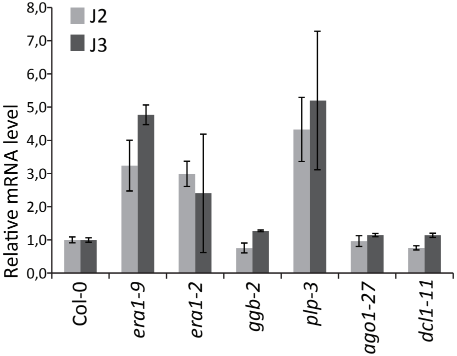

Figure S6 J2 and J3 mRNA is induced in FTase mutants

Total RNA from 16-day old seedlings was subjected to oligo(dT)-primed reverse transcription, and J2 and J3 mRNAs were quantified by real-time PCR using actin as a normalization control. Error bars indicate standard deviations among technical triplicates.

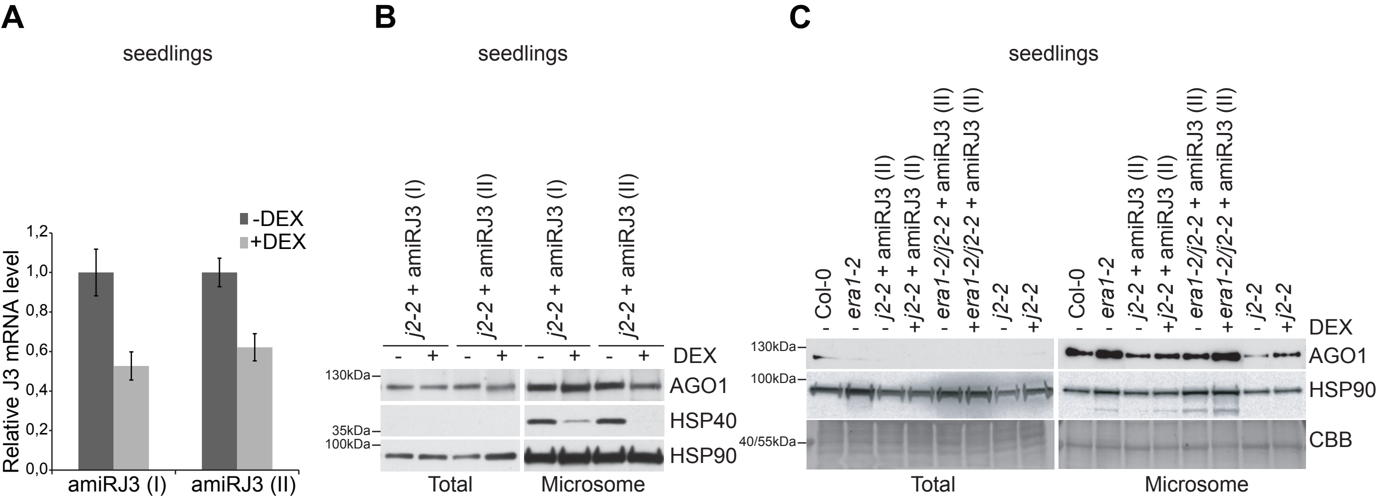

Figure S7 Analysis of AGO1 levels in membrane fraction upon J2/J3 knock-down

(A) Quantitative RT-PCR analysis of J3 mRNA in Col-0 seedlings expressing the dexamethasone inducible artificial miRNA targeting J3 (amiR-J3). amiR-J3(I) and (II) indicate two independent lines expressing amiR-J3. Error bars represent standard deviation between triplicates.

(B-C) Western blot of AGO1, J2/J3, and HSP90 in total and microsome fractions prepared from lysates of 16-day old seedlings of the indicated genotypes grown in the presence of dexamethasone to induce the expression of the artificial miRNA. Proteins were extracted from equal amount of plant tissue, and the same volume was loaded on the gel was each sample. Coomassie Brilliant Blue (CBB), was used to stain the membranes in C. These loading controls panels come from different sections of the full membranes: the leftmost molecular weight marker corresponds to the membrane with total lysates, the rightmost molecular weight marker corresponds to the membrane with microsome fractions.

Oligonucleotide #	Oligonucleotide name	Sequence 5'-3'	Use
1	RP-era1-C-Fw	CCTAGGAGATTACATCTTGAG	Genotype era1-9
2	LP-era1-C-Rv	CAATTTGTTCGTCCTACCTTG	Genotype era1-9
3	LB1*	GCCTTTTCAGAAATGGATAAATAGCCTTGCTTCC	Genotype era1-9
4	era1-06-fw	AAGTCTGGACCCATTATGCTA	Genotype era1-2
5	era1-08-rv	CATCATCATCACTGTCTTCAA	Genotype era1-2
6	plp-C09-Fw	AAACGAGAGCTCATGGCGATA	Genotype plp-3
7	plp-C09-Rv	CTGCCACTGTAATCTTGCTCT	Genotype plp-3
8	GABI_O8760-Rv*	GGGCTACACTGAATTGGTAGCTC	Genotype plp-3
9	LP2-J2-d2-Fw	GAAGAGGACCTTCAAGGAAGA	Genotype j2-2
10	RP2-J2-d2-Rv	TCATAAGCCTGTGCTAACTCT	Genotype j2-2
11	J3-2-TDNA-Fw	ATATACCAGAGGCCATTCATG	Genotype j3-2
12	J3-UTR-TDNA-Rv	TCACTATAAATTAATAAAATTGCG	Genotype j3-2
13	LBb1.3*	ATTTTGCCGATTTCGGAAC	Genotype j2-2 and j3-2
14	SAQQ-PfeI-Fw	GGTGGTGCTCAAAGGGTGGAAT	Genotype J3C417S
15	SAQQ-PfeI-Rv	GCAAGAGACAAATTGGTTGGAGC	Genotype J3C417S
16	miR156 probe	GTGCTCACTCTCTTCTGTCA	Northern hybridization
17	miR159 probe	TAGAGCTCCCTTCAATCCAAA	Northern hybridization
18	miR160 probe	TGGCATACAGGGAGCCAGGCA	Northern hybridization
19	miR167 probe	TAGATCATGCTGGCAGCTTCA	Northern hybridization
20	miR166 probe	GGGGAATGAAGCCTGGTCCGA	Northern hybridization
21	miR398 probe	CAGGGGTGACCTGAGAACACA	Northern hybridization
22	miR403 probe	AATTAGATTCAAGCACAAAACA	Northern hybridization
23	U6 probe	AGGGGCCATGCTAATCTTCTC	Northern hybridization
24	PHB_FWD_qPCR	AGAGTTCCTTTCCAAGGCTACAG	PHB qPCR
25	PHB_REV_qPCR	ATAGCGACTATGCCAATAGAATCC	PHB qPCR
26	REV_qPCR_Fw1	TAC ACA GCT GAG CAA GTC GA	REV qPCR
27	REV_qPCR_Rv1	GTT GTC GAC GGA GAG AGC TA	REV qPCR
28	MYB65_FWD_qPCR	GATGGTTCCTGATAGCCATACAGTTAC	MYB65 qPCR
29	MYB65_REV_qPCR	TAGGCATCAACAGAGTCAAGGAGATC	MYB65 qPCR
30	YUC5_qPCR_Fw	TTC CAG AGA TCG ATG GGC TC	YUC5 qPCR
31	YUC5_qPCR_Rv	TCC ACA TCC GAC GAC AAG AA	YUC5 qPCR
32	IAA20_qPCR_Fw1	TGT CTC TTA ATG GCT ACC GCG ACT	IAA20 qPCR
33	IAA20_qPCR_Rv1	TCA GCC CAG AGA ATG GAT GCG T	IAA20 qPCR
34	IAA30_qPCR_Fw1	TTC AAT GCT TCA ATC CTT TGG	IAA30 qPCR
35	IAA30_qPCR_Rv1	AGC ACG TGA CTC TTC TCA CTA CA	IAA30 qPCR
36	CP1 qt F	CAAGGAACTTGCGGAAGTTGT	CP1 qPCR
37	CP1 qt R	CGTAGTCCATTAAACCGCCG	CP1 qPCR
38	TAA1_qPCR_Fw	TGG AGA ACT CGA GGA AAC CC	TAA1 qPCR
39	TAA1_qPCR_Rv	CAC CTG TCA CCC ATC TTC CT	TAA1 qPCR
40	AGO1_FWD_qPCR	AAGGAGGTCGAGGAGGGTATGG	AGO1 qPCR
41	AGO1_REV_qPCR	CAAATTGCTGAGCCAGAACAGTAGG	AGO1 qPCR
42	SPL3_FWD_qPCR	CAAGTAGTAGTGGAGTTTGTCAGGTCG	SPL3 qPCR
43	SPL3_REV_qPCR	TTTCCGCCTTCTCTCGTTGTGTCC	SPL3 qPCR
44	TIR1_FWD_qPCR	GCCTCTCTCTATCTGGCCTCTT	TIR1 qPCR
45	TIR1_REV_qPCR	AGGGCAGCTCTCTGGTCTCGA	TIR1 qPCR
46	ARF8_FWD_qPCR	AGATGTTTGCTATCGAAGGGTTGTTG	ARF8 qPCR
47	ARF8_REV_qPCR	CCATGGGTCATCACCAAGGAGAAG	ARF8 qPCR
48	CIP4_FWD_qPCR	CAGTGAGTTGACATCTACTCCAGTTAC	CIP4 qPCR
49	CIP4_REV_qPCR	CGTTCACAATTTCTCTTGAAGC	CIP4 qPCR
50	CSD2_FWD_qPCR	TTTCATCTCCATGAGTTTGGTG	CSD2 qPCR
51	CSD2_REV_qPCR	AAAGGCTCTTCCAACAACAGA	CSD2 qPCR
52	AGO2_qPCR_Fw	TAACTCCTTTTTTCTGGTAGA	AGO2 qPCR
53	AGO2_qPCR_Rv	ACCAAAAAAAGAAGAAGACGT	AGO2 qPCR
54	TAS1B_FWD_qPCR	CCATGTGTCAGTTTCGACCA	TAS1B qPCR
55	TAS1B_REV_qPCR	GGTGAATGGTTAGATACCGATGA	TAS1B qPCR
56	PHV_FWD_qPCR	CGTGATGTTAACAACCCAGCTA	PHV qPCR
57	PHV_REV_qPCR	CGTGAAACAGCTACGATACCAA	PHV qPCR
58	SCL6-IV_FWD_qPCR	TGTCTAGCTCAGGGGATATTGG	SCL6-IV qPCR
59	SCL6-IV_REV_qPCR	AGCTGCTCTTTCTAATGGCTTC	SCL6-IV qPCR
60	J3-qPCR-1-Fw	GAGGCCCCTTTGGAGGTA	J3 qPCR
61	J3-qPCR-1-Rv	GGATGAACAACATCCTCACCA	J3 qPCR
62	J2-qPCR-1-Fw	GGTAGTGGTGGACACCCATTC	J2 qPCR
63	J2-qPCR-1-Rv	GGATGAACAACATCtTCACCA	J2 qPCR
64	J3-qPCR-2-Fw	TCCTTAGAGAGACTTTGACCCA	J3 qPCR
65	J3-qPCR-2-Rv	AACAAGTTTCGATGTTCCACCG	J3 qPCR
66	J2-qPCR-2-Fw	CAACATGCGCATCTTAGTGATC	J2 qPCR
67	J2-qPCR-2-Rv	GAATTAGGACGAGGTTCTTCC	J2 qPCR
68	SUL-fwd probe	ATATCGAAAAGGCTTTGACAGAAG	Northern hybridization
69	SUL-rev probe	AATCTGGTCTTGAAGCTTGTCC	Northern hybridization
70	UBQ10(U)_fwd	GGCTTAATGTGCTTTCCTTACATTCTGAGCC	USER cloning: UL403 and UL403m
71	UBQ10(U)_FLuc_rev	ATCCAATCUGTTAATCAGAAAAACTCAG	USER cloning: UL403 and UL403m
72	FLuc(U)_UBQ10_fwd	AGATTGGAUCCACCATGGAAGACGCC	USER cloning: UL403 and UL403m
73	FLUC(U)_RV	ACAATTUGGACTTTCCGCCCTTCTT	USER cloning: UL403 and UL403m
74	AGO2_3'UTR(U)_FW	AAATTGUGAAGAAGAGAGTGAGTTT	USER cloning: UL403 and UL403m
75	403mut(U)_RV	ATCTAAGTUGCACAAACTCCTTTCTACCA	USER cloning: UL403 and UL403m
76	403mut(U)_FW	AACTTAGAUATTGGGTTTTTCGTAGTG	USER cloning: UL403 and UL403m
77	AGO2_3'UTR(U)_RV	GGTTTAAUACCAAAAAAAGAAGAAGAC	USER cloning: UL403 and UL403m

Supplementary Table S1: Oligonucleotydes used in this study

Table S2

Accession	Mutant	Transgene	Insertion	Reference
Col-0	*era1-2*			(4)
Ler	*era1-4*			(5)
Col-0	*era1-9*		SAIL_146D09	(6,7)
Col-0	*ggb-1*			(8)
Col-0	*plp-3*		GABI-KAT 386C07	(7,9)
Col-0	*ago1-27*			(10)
Col-0	*ago1-38*			(11)
Col-0	*dcl1-11*			(12)
Col-0	*dcl1-11/era1-2*			This work
Col-0	*ago1-27/era1-2*			This work
Col-0	*hsp90.2-3*			(13)
Col-0	*hsp90.2-3/era1-2*			This work
Col-0		SUC:SUL		(14)
Col-0	*era1-2*	SUC:SUL		This work
Col-0	*plp-3*	SUC:SUL	GABI-KAT 386C07	This work
Col-0	*j2-2/j3-2*	SUC:SUL + J3(WT)/J3(C417S)		This work
Col-0	*j2-2/j3-2*	SUC:SUL + J3(WT)/J3(C417S)		This work
Col-0		pJ3:2xFLAG-2xHA-J3		(7)
Col-0		pJ3:2xFLAG-2xHA-J3(C417S)		(7)
Col-0	*j3-1*		SALK_132923	(15)
Col-0	*j3-2*		SALK_141625	(7,16)
Col-0	*j2-2*		SALK_071563	(7,16)
Col-0	*j2-2*	pDEX:amiR-J3		(7)
Col-0	*j2-2/era1-2*	pDEX:amiR-J3		This work
Col-0	*j3-1*	pJ3:J3		(7)
Col-0	*j3-1*	pJ3:J3(C417S)		(7)
Col-0	*j2-2/j3-2*	pJ3:J3		(7)
Col-0	*j2-2/j3-2*	pJ3:J3(C417S)		(7)
Col-0	*j3-1/plp-3*			This work
Col-0		SPL9:GUS/SPL9:GUSm		(17)
Col-0	*era1-2*	SPL9:GUS/SPL9:GUSm		This work
Col-0	*plp-3*	SPL9:GUS/SPL9:GUSm	GABI-KAT 386C07	This work
Col-0		GFP171.14		(1)
Col-0	*era1-2*	GFP171.14		This work
Col-0		UL403/UL403m		This work
Col-0	*era1-2*	UL403/UL403m		This work

Table S2. Arabidopsis mutants and transgenic lines used in this study.

Table S3

Antibody	Organism/type/antigen	Reference
AGO1 (for IP)	Rabbit/polyclonal	Agrisera AS09 527
AGO1 (western)	Rabbit/polyclonal/peptide	This work
ChlI (SUL)	Rabbit/polyclonal/Synechosystis ChlI protein	(18), (19)
Hsp40 (J2/J3)	Rabbit/polyclonal/peptide	This work
Hsp70	Rabbit/polyclonal	Agrisera AS08 371
Hsp90-1	Rabbit/polyclonal	Agrisera AS08 346
FLAG	Mouse/monoclonal/peptide	Sigma A8592
SIP2	Rabbit/polyclonal	Agrisera AS09 496
BiP	Rabbit/polyclonal	Agrisera AS09 481
ARF1	Rabbit/polyclonal	Agrisera AS08 325
H+/ATPase	Rabbit/polyclonal	Agrisera AS07 260
V/ATPase	Rabbit/polyclonal	Agrisera AS09 577
VDAC1	Rabbit/polyclonal	Agrisera AS07 212
PEX14	Rabbit/polyclonal	Agrisera AS08 372

Table S3. Antibodies used in this study

References

1. Moissiard, G., Parizotto, E. A., Himber, C., and Voinnet, O. (2007) Transitivity in Arabidopsis can be primed, requires the redundant action of the antiviral Dicer-like 4 and Dicer-like 2, and is compromised by viral-encoded suppressor proteins. *RNA* **13**, 1268-1278
2. Parizotto, E. A., Dunoyer, P., Rahm, N., Himber, C., and Voinnet, O. (2004) In vivo investigation of the transcription, processing, endonucleolytic activity, and functional relevance of the spatial distribution of a plant miRNA. *Genes & development* **18**, 2237-2242
3. Wu, G., Park, M. Y., Conway, S. R., Wang, J.-W., Weigel, D., and Poethig, R. S. (2009) The Sequential Action of miR156 and miR172 Regulates Developmental Timing in Arabidopsis. *Cell* **138**, 750-759
4. Cutler, S., Ghassemian, M., Bonetta, D., Cooney, S., and McCourt, P. (1996) A protein farnesyl transferase involved in abscisic acid signal transduction in Arabidopsis. *Science* **273**, 1239-1241
5. Ziegelhoffer, E. C., Medrano, L. J., and Meyerowitz, E. M. (2000) Cloning of the Arabidopsis WIGGUM gene identifies a role for farnesylation in meristem development. *Proc Natl Acad Sci USA* **97**, 7633-7638
6. Sessions, A., Burke, E., Presting, G., Aux, G., McElver, J., Patton, D., Dietrich, B., Ho, P., Bacwaden, J., Ko, C., Clarke, J. D., Cotton, D., Bullis, D., Snell, J., Miguel, T., Hutchison, D., Kimmerly, B., Mitzel, T., Katagiri, F., Glazebrook, J., Law, M., and Goff, S. A. (2002) A high-throughput Arabidopsis reverse genetics system. *Plant Cell* **14**, 2985-2994
7. Barghetti, A., Sjogren, L., Floris, M., Paredes, E. B., Wenkel, S., and Brodersen, P. (2017) Heat-shock protein 40 is the key farnesylation target in meristem size control, abscisic acid signaling, and drought resistance. *Genes & Development* **31**, 2282-2295
8. Johnson, C. D., Chary, S. N., Chernoff, E. A., Zeng, Q., Running, M. P., and Crowell, D. N. (2005) Protein geranylgeranyltransferase I is involved in specific aspects of abscisic acid and auxin signaling in Arabidopsis. *Plant Physiol* **139**, 722-733
9. Rosso, M. G., Li, Y., Strizhov, N., Reiss, B., Dekker, K., and Weisshaar, B. (2003) An Arabidopsis thaliana T-DNA mutagenized population (GABI-Kat) for flanking sequence tag-based reverse genetics. *Plant Mol Biol* **53**, 247-259

10. Morel, J.-B., Gordon, C., Mourrain, P., Beclin, C., Boutet, S., Feuerbach, F., Proux, F., and Vaucheret, H. (2002) Fertile hypomorphic *ARGONAUTE* (*ago1*) mutants impaired in post-transcriptional gene silencing and virus resistance. *Plant Cell* **14**, 629-639
11. Gregory, B. D., O'Malley, R. C., Lister, R., Urich, M. A., Tonti-Filippini, J., Chen, H., Millar, A. H., and Ecker, J. R. (2008) A link between RNA metabolism and silencing affecting Arabidopsis development. *Dev Cell* **14**, 854-866
12. Zhang, J. F., Yuan, L. J., Shao, Y., Du, W., Yan, D. W., and Lu, Y. T. (2008) The disturbance of small RNA pathways enhanced abscisic acid response and multiple stress responses in Arabidopsis. *Plant Cell Environ* **31**, 562-574
13. Hubert, D. A., Tornero, P., Belkhadir, Y., Krishna, P., Takahashi, A., Shirasu, K., and Dangl, J. L. (2003) Cytosolic HSP90 associates with and modulates the Arabidopsis RPM1 disease resistance protein. *EMBO J* **22**, 5679-5689
14. Himber, C., Dunoyer, P., Moissiard, G., Ritzenthaler, C., and Voinnet, O. (2003) Transitivity-dependent and -independent cell-to-cell movement of RNA silencing. *EMBO J* **22**, 4523-4533
15. Shen, L., Kang, Y. G., Liu, L., and Yu, H. (2011) The J-domain protein J3 mediates the integration of flowering signals in Arabidopsis. *Plant Cell* **23**, 499-514
16. Alonso, J. M., Stepanova, A. N., Leisse, T. J., Kim, C. J., Chen, H., Shinn, P., Stevenson, D. K., Zimmerman, J., Barajas, P., Cheuk, R., Gadrinab, C., Heller, C., Jeske, A., Koesema, E., Meyers, C. C., Parker, H., Prednis, L., Ansari, Y., Choy, N., Deen, H., Geralt, M., Hazari, N., Hom, E., Karnes, M., Mulholland, C., Ndubaku, R., Schmidt, I., Guzman, P., Aguilar-Henonin, L., Schmid, M., Weigel, D., Carter, D. E., Marchand, T., Risseeuw, E., Brogden, D., Zeko, A., Crosby, W. L., Berry, C. C., and Ecker, J. R. (2003) Genome-wide insertional mutagenesis of Arabidopsis thaliana. *Science* **301**, 653-657
17. Yang, L., Conway, S. R., and Poethig, R. S. (2011) Vegetative phase change is mediated by a leaf-derived signal that represses the transcription of miR156. *Development* **138**, 245-249
18. Petersen, B. L., Jensen, P. E., Gibson, L. C., Stummann, B. M., Hunter, C. N., and Henningsen, K. W. (1998) Reconstitution of an active magnesium chelatase enzyme complex from the bchI, -D, and -H gene products of the green sulfur bacterium Chlorobium vibrioforme expressed in Escherichia coli. *J Bact* **180**, 699-704
19. Brodersen, P., Sakvarelidze-Achard, L., Bruun-Rasmussen, M., Dunoyer, P., Yamamoto, Y. Y., Sieburth, L., and Voinnet, O. (2008) Widespread translational inhibition by plant miRNAs and siRNAs. *Science* **320**, 1185-1190
